# Iridoid Glycosides and Coumarin Glycoside Derivatives from the Roots of *Nymphoides peltata* and Their In Vitro Wound Healing Properties

**DOI:** 10.3390/ijms25021268

**Published:** 2024-01-19

**Authors:** Tae-Young Kim, Bum Soo Lee, Beom-Geun Jo, Seong Pil Heo, Young Suk Jung, Su-Nam Kim, Ki Hyun Kim, Min Hye Yang

**Affiliations:** 1 Department of Pharmacy, College of Pharmacy and Research Institute for Drug Development, Pusan National University, Busan 46241, Republic of Korea; taeyour@pusan.ac.kr (T.-Y.K.); bg_jo@pusan.ac.kr (B.-G.J.);; 2 School of Pharmacy, Sungkyunkwan University, Suwon 16419, Republic of Korea; kosboybs@skku.edu; 3 Natural Products Research Institute, Korea Institute of Science and Technology, Gangneung 25451, Republic of Korea; 123045@kist.re.kr (S.P.H.); snkim@kist.re.kr (S.-N.K.); 4 Division of Bio-Medical Science and Technology, KIST School, University of Science and Technology, Seoul 02792, Republic of Korea

**Keywords:** *Nymphoides peltata*, peltatamarin A, peltatamarin B, human keratinocyte cell, wound healing, cell migration, cell proliferation

## Abstract

*Nymphoides peltata* has been used as a medicinal herb in traditional medicines to treat strangury, polyuria, and swelling. The phytochemical investigation of the MeOH extract of *N. peltata* roots led to the isolation of three iridoid glycosides and three coumarin glycoside derivatives, which were characterized as menthiafolin (**1**), threoninosecologanin (**2**), callicoside C (**3**), and scopolin (**4**), as well as two undescribed peltatamarins A (**5**) and B (**6**). The chemical structures of the undescribed compounds were determined by analyzing their 1 dimensional (D) and 2D nuclear magnetic resonance (NMR) spectra and using high-resolution (HR)-electrospray ionization mass spectroscopy (ESI-MS), along with the chemical reaction of acid hydrolysis. The wound healing activities of the isolated compounds **1**–**6** were evaluated using a HaCaT cell scratch test. Among the isolates, scopolin (**4**) and peltatamarin A (**5**) promoted HaCaT cell migration over scratch wounds, and compound **5** was the most effective. Furthermore, compound **5** significantly promoted cell migration without adversely affecting cell proliferation, even when treated at a high dose (100 μM). Our results demonstrate that peltatamarin A (**5**), isolated from *N. peltata* roots, has the potential for wound healing effects.

## 1. Introduction

The skin is the body′s outermost organ and its first line of defense against external factors [1]. However, the structural integrity of the skin can be compromised by physical and chemical factors and result in functional impairments [2]. Wound healing is a complex process that involves the repair of damaged tissue and typically occurs in four distinct phases: hemostasis, inflammation, proliferation, and remodeling [3,4]. In particular, the inflammatory phase is a key determinant of whether the wound healing process is delayed or accelerated [5]. During this phase, inflammatory cells such as neutrophils, monocytes, macrophages, T-lymphocytes, and mast cells are recruited to wounds by vasodilation [6]. Neutrophils are the first inflammatory cells to arrive at wounds and are responsible for protection from bacterial infection and perpetuating the early inflammatory stage by secreting pro-inflammatory cytokines [7]. Simultaneously, monocytes from blood are attracted to wounds and differentiate into macrophages, which produce an array of growth factors and cytokines and facilitate re-epithelialization and angiogenesis [8]. During the later inflammatory stage, lymphocytes and mast cells participate in the remodeling phase and accelerate wound healing [9,10]. Recently, various studies have been conducted to identify plant secondary metabolites with therapeutic or cosmetic potential with limited side effects compared to commercial drugs that promote skin regeneration [11,12,13].

Menyanthaceae is a family of perennial aquatic plants that consists of about 60 species in six genera [14]. The genus *Nymphoides* is the largest in Menyanthaceae and contains about 55 species [15]. Plants of this species are native to temperate and subtropical regions and are endemic in Korea, China, and Japan [16,17]. Phytochemical studies on the *Nymphoides* genus have revealed that they contain polyphenols, flavonoids, triterpenes, and ferulic acid as major compounds, and that their pharmacological activities include anticonvulsant, antioxidant, and anti-diabetic effects [18,19,20,21]. The species *Nymphoides peltata,* also known as the yellow floating heart, is a medicinal herb used in traditional Chinese medicines, such as Ben Cao Gang Mu, and in traditional Indian medicines, such as Ayurveda, and is used to treat heat strangury, polyuria, diuretic, antipyretic, and swelling [18]. In a recent pharmacological study, an MTT assay revealed a 95% ethanol extract of *N. peltata* exhibited significant antitumor activity against prostate cancer (PC3) and osteosarcoma cells (U2OS) [22]. Also, our recent studies showed that a 95% ethanol (EtOH) extract of *N. peltata* root inhibited IL-4 expression in PMA/ionomycin-induced RBL-2H3 cells and had anti-atopic effects in oxazolone- and 2,4-dinitrochlorobenzene (DNCB)-induced mouse models [23]. Although many studies have reported the pharmacological activity of *N. peltata* extract, little research has been conducted on its phytochemical composition at the species level. In this study, we isolated and identified three iridoid glycosides (**1**–**3**) and three coumarin glycoside derivatives (**4**–**6**), including two new coumarin glycosides (**5** and **6**) from the MeOH extract of *N. peltata* root. The chemical structures of the new compounds were determined by analyzing their 1 dimensional (D) and 2D nuclear magnetic resonance (NMR)spectra and using high-resolution (HR)-electrospray ionization mass spectroscopy (ESI-MS), along with the chemical reaction of acid hydrolysis. Herein, we have described the isolation and structural characterization of the compounds (**1**–**6**) and the evaluation of their potential wound healing effects using an in vitro human keratinocyte scratch model.

## 2. Results and Discussion

### 2.1. Isolation of the Compounds

**Figure 1:**
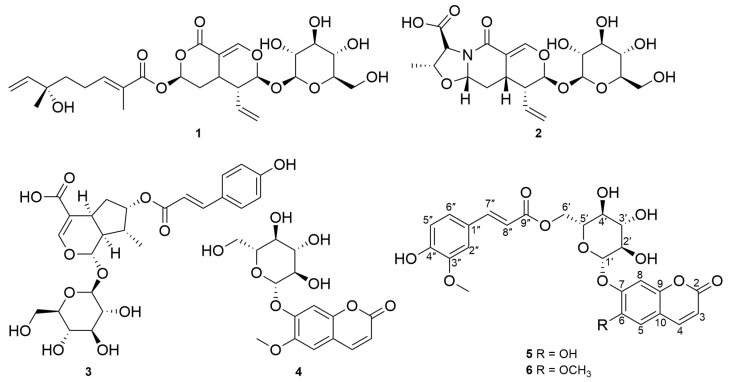
The chemical structures of the isolated compounds **1**–**6**.

The roots of *N. peltata* were extracted with methanol (MeOH), and the resulting MeOH extract was fractionated by solvent-partitioning with *n*-hexane (Hx), ethyl acetate (EtOAc), and *n*-butanol (BuOH) to obtain three major fractions. Column chromatography procedures, including open-column chromatography, Sephadex LH-20, and reverse-phase HPLC purification, were applied to the EtOAc and *n*-BuOH fractions, which led to the isolation of six compounds. The isolated compounds include three iridoid glycosides (**1**–**3**) and three coumarin derivatives (**4**–**6**). The chemical structures of the isolated compounds were definitively determined to be menthiafolin (**1**) [20], threoninosecologanin (**2**) [24], callicoside C (**3**) [25], and scopolin (**4**) [26] through the comparison and validation of their NMR spectroscopic data (Figures S1–S4) with those previously reported and high-resolution electrospray ionization mass spectrometry (HR-ESI-MS) analyses (Figure 1 and Table S1). Among the isolated compounds, two compounds, **5** and **6,** were identified as new coumarin glycoside derivatives.

### 2.2. Structural Elucidation of the New Compounds

**Figure 2:**
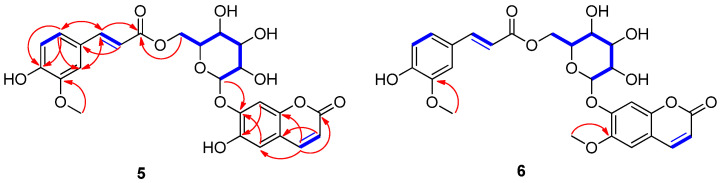
Key ^1^H-^1^H COSY (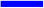) and HMBC (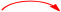) correlations for compounds **5** and **6**.

Compound **5** was isolated as a pale brown solid. Its molecular formula was determined as C_25_H_24_O_12_ from the proton-adducted molecular ion at *m*/*z* 539.1138 [M + Na]^+^ (calculated for C_25_H_24_O_12_Na, 539.1165) in the positive-ion mode of HR-ESI-MS data (Figure S5). The IR spectrum exhibited absorptions corresponding to hydroxyl (3385 cm^−1^) and carbonyl (1705 cm^−1^) functionalities. The ^1^H NMR (Table 1) spectrum of compound **5** exhibited the presence of proton signals attributable to four olefinic double bonds at *δ*_H_ 6.22 (1H, d, *J* = 9.5 Hz, H-3)/*δ*_H_ 7.85 (1H, d, *J* = 9.5 Hz, H-4) and *δ*_H_ 6.39 (1H, d, *J* = 16.0 Hz, H-8″)/*δ*_H_ 7.47 (1H, d, *J* = 16.0 Hz, H-7″), corresponding to *cis* and *trans* double bonds, respectively; characteristic 1,3,4-trisubstituted benzene signals at *δ*_H_ 6.72 (1H, d, *J* = 8.0 Hz, H-5″), 7.03 (1H, dd, *J* = 8.0 and 2.0 Hz, H-6″), and 7.21 (1H, d, *J* = 2.0 Hz, H-2″); two singlet aromatic protons at *δ*_H_ 7.04 (1H, s, H-5) and 7.10 (1H, s, H-8); and one methoxy group at *δ*_H_ 3.76 (3H, s); as well as one characteristic anomeric proton signal at *δ*_H_ 5.02 (1H, d, *J* = 7.5 Hz, H-1′) (Figure S6). The ^13^C NMR (Table 1) data of **5**, obtained with the assistance of HSQC (Figure S9) and HMBC spectra, confirmed the existence of twenty-five carbon signals including two carbonyl carbons at *δ*_C_ 160.9 (C-2) and 167.1 (C-9″); four olefinic double bonds at *δ*_C_ 113.9 (C-3), 114.3 (C-8″), 144.5 (C-4), and 145.9 (C-7″); characteristic 1,3,4-trisubstituted benzene carbon signals at *δ*_C_ 111.8 (C-2″), 116.0 (C-5″), 123.5 (C-6″), 125.9 (C-1″), 148.4 (C-3″), and 149.9 (C-4″); two characteristic methine aromatic carbons at *δ*_C_ 103.6 (C-8) and 113.2 (C-5); one methoxy carbon at *δ*_C_ 56.1; and six oxygenated carbons, corresponding to glucose moiety, at *δ*_C_ 63.8 (C-6′), 70.4 (C-4′), 73.5 (C-2′), 74.3 (C-5′), 76.2 (C-3′), and 100.9 (C-1′) (Figure S7). A detailed inspection of the ^1^H and ^13^C NMR data of **5** revealed that the NMR data closely resembled those of 6′-*O*-caffeoyl scopoline [27], which is a coumarin glycoside derivative. The only difference was in the chemical shifts of the 1,3,4-trisubstituted benzene carbon signals, which implied a different location of the methoxy group compared to 6′-*O*-caffeoyl scopolin [27]. Using ^1^H-^1^H COSY (Figure S8) and HMBC correlations (Figure S10), the gross structure of compound **5** was confirmed. The HMBC correlations from H-4 to C-2 (*δ*_C_ 160.9), from H-5 to C-7 (*δ*_C_ 149.1)/C-9 (*δ*_C_ 148.3), and from H-8 to C-6 (*δ*_C_ 144.0)/C-10 (*δ*_C_ 113.5) indicated the presence of the coumarin moiety (Figure 2). Moreover, HMBC correlations from H-7″ to C-6″ (*δ*_C_ 123.5)/C-2″ (*δ*_C_ 111.8)/C-9″ (*δ*_C_ 167.1), from H-8″ to C-1″ (*δ*_C_ 125.9), from H-6″ to C-4″ (*δ*_C_ 149.9)/C-2″ (*δ*_C_ 111.8)/C-7″ (*δ*_C_ 145.9), from H-5″ to C-3″ (*δ*_C_ 148.4), from H-2″ to C-6″ (*δ*_C_ 123.5)/C-4″ (*δ*_C_ 149.9)/C-7″ (*δ*_C_ 145.9), and from the methoxy group to C-3″ clearly indicated the presence of a feruloyl group (Figure 2). Finally, the HMBC correlations from H-1′ to C-7 (*δ*_C_ 149.1) and from H-6′ to C-9″ (*δ*_C_ 167.1) proved the connection between the coumarin moiety, glucose, and the feruloyl group (Figure 2). The coupling constant (*J* = 7.5 Hz) of the anomeric proton at *δ*_H_ 5.02 confirmed the presence of *β*-glucopyranose, and acid hydrolysis of compound **5** produced D-glucopyranose [28]. Accordingly, compound **5** was determined as 6-hydroxy-coumarin-7-*O*-(6′-*O*-feruloyl)-*β*-D-glucopyranoside, as shown in Figure 2, and named peltatamarin A.

Compound **6** was isolated as a pale brown solid. The molecular formula was confirmed to be C_26_H_26_O_12_ from the molecular ion peak at *m*/*z* 553.1324 [M + Na]^+^ (calculated for C_26_H_26_O_12_Na, 553.1322) using positive-ion HR-ESI-MS (Figure S12). The ^1^H and ^13^C NMR (Table 1 and Figures S13 and S14) of **6**, obtained with the assistance of HSQC (Figure S16) and HMBC spectra (Figure S17), were almost identical to those of **6**, except for the chemical shifts of an additional methoxy group at *δ*_H_ 3.81 (3H, s) and *δ_C_* 56.5). The locations of the two methoxy groups in compound **6** were clearly assigned at C-6 and C-3″ by the HMBC experiment, where the HMBC correlations between methoxy group at *δ*_H_ 3.81 and C-6 (*δ_C_* 146.2) and between methoxy group at *δ*_H_ 3.79 and C-3″ (*δ_C_* 148.5) provided critical information for the locations of methoxy groups (Figure 2). Finally, the gross structure of compound **6** was unambiguously confirmed by the analysis of the ^1^H-^1^H COSY (Figure S15) and HMBC correlations (Figure 2). Therefore, the chemical structure of **6** was elucidated to be 6-methoxy-coumarin-7-*O*-(6′-*O*-feruloyl)-*β*-D-glucopyranoside, as shown in Figure 2, and named peltatamarin B.

### 2.3. Evaluation of Biological Activity of the Isolated Compounds

**Figure 3:**
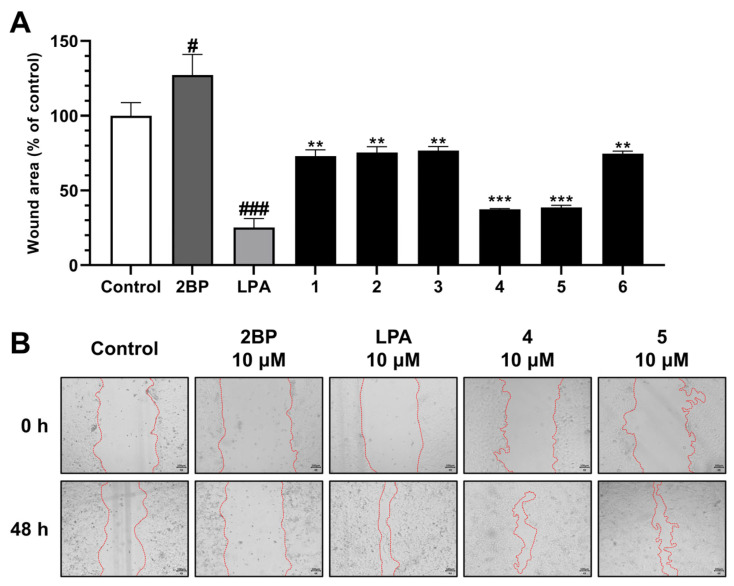
Effect of compounds **1**–**6** on HaCaT cell wound healing assay results. Scratched HaCaT monolayers were treated with 10 μM of 2BP, LPA, or compounds **1**–**6**, and cell migration was observed under a microscope after 48 h of culture (**A**). Photographs of a wounded area of compounds **4** and **5** were captured using a 4× objective lens for a microscope camera (scale bar = 100 µm) (**B**). Each bar is presented as mean ± SD of three independent experiments. # *p* < 0.05 and ### *p* < 0.001 vs. CON group; ** *p* < 0.01 and *** *p* < 0.001 vs. 2BP group.

Keratinocytes are constituents of the epidermis and play a pivotal role in the regeneration of the epidermis during wound healing (hemostasis, inflammation, proliferation, and remodeling) [29,30]. The wound healing activities of compounds **1**–**6** from *N. peltata* were evaluated using a HaCaT cell scratch test. This in vitro test is useful for evaluating cell migration from wound edges over scratches [31]. Treatment with 2**-**bromo-palmitate (2BP, the negative control) reduced cell migration and increased wound area to 127.31%. Lysophosphatidic acid (LPA), which contributes to epidermal regeneration by modulating cellular responses [32], was used as the positive control and the reduced wound area to 25.32%. Furthermore, our results revealed that HaCaT cells showed increased wound healing in the presence of scopolin (**4**) (to 37.46%) or peltatamarin A (**5**) (to 38.67%) groups (Figure 3 and Figure S19).

Previous studies have shown that the bioactivities of various coumarins are influenced by the types of substituents at C-6 and/or C-7 [33,34,35]. 6,7-OH coumarin had better anti-inflammatory and antioxidant activities than 6-OCH_3_ and 7-OH coumarin [33,34]. Moreover, a coumarin glycoside with a 6-OH group on the coumarin scaffold had a more potent anti-diabetic effect than the glycoside with a 6-OCH_3_ substitution [36]. These results improved our understanding of the structure–activity relationships of compound **5** compared to compound **6**. In the present study, compound **5** showed a two-fold increase in wound healing versus compound **6**.

**Figure 4:**
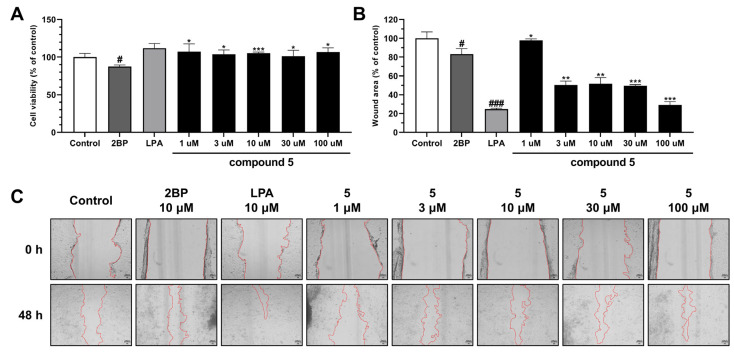
Effects of compound **5** on HaCaT cells as determined by MTT and wound healing assays. Scratched HaCaT cell monolayers were treated with 10 μM of 2BP; 10 μM of LPA; or 1, 3, 10, 30, or 100 μM of compound **5** for 48 h. Cell proliferation was evaluated using an MTT assay (**A**), and cell migration was evaluated using the wound healing assay (**B**). Photographs of a wounded area were captured using a 4× objective lens for a microscope camera (scale bar = 100 µm) (**C**). Each bar is presented as mean ± SD of three independent experiments. # *p* < 0.05 and ### *p* < 0.001 vs. CON group; * *p* < 0.05, ** *p* < 0.01, and *** *p* < 0.001 vs. 2BP group.

The effects of compound **5** on HaCaT cell proliferation and migration were also investigated at various concentrations (1, 3, 10, 30, or 100 μM). Interestingly, compound **5** at 1, 3, 10, 30, or 100 μM had no significant effect on cell proliferation as determined by an MTT assay. On the other hand, the wound healing analysis showed treating HaCaT cells with 10 μM LPA (the positive control) reduced the wound area to 24.60% versus the non-treated controls (100%) (Figure 4A). However, treatment with compound **5** at 3, 10, or 30 μM reduced wound areas to about 50%, and at 100 μM, it reduced wound area to 29.17% (Figure 4B,C). Skin damage induces the migration of immune cells to the site of injury, and these cells then secrete inflammatory species and growth factors [29,37].

Furthermore, this process, is facilitated by inflammatory cells and growth factors and plays an important role in wound healing by increasing cell proliferation and restoring damaged tissues [37,38]. Therefore, our results suggest that compound **5** has potential use as a wound healing promoter and that it does so by enhancing cell migration but not cell proliferation.

## 3. Materials and Methods

### 3.1. General Experimental Procedures

The instrument used for nuclear magnetic resonance (NMR) spectra analysis (^1^H, ^13^C, ^1^H-^1^H COSY, HMBC, and HMQC) were observed using a JEOL JNM-ECZ400S 400 MHz NMR spectrometer (JEOL, Ltd., Tokyo, Japan) and Bruker AVANCE NEO 500 MHz NMR spectrometer (Bruker Corp., Billerica, MA, USA). The instrument used for HR ESI-MS was an Agilent 6530 Accurate-Mass Q/TOF-LC/MS system (Agilent Technologies, Santa Clara, CA, USA). The high-performance liquid chromatography-photodiode array (HPLC-PDA) analysis was performed on the Waters HPLC system with an e2695 separation module and a 2998 PDA detector (Waters Corp., Milford, MA, USA) and Empower version^®^3 Chromatography Software (Build 3471, Waters Corp., MA, USA) using a reversed phase column (Aegispak C18-L, 5 μm, 4.6 × 250 mm, Young Jin Biochrom., Sungnam, Republic of Korea). Semi-preparative HPLC was performed on a Gilson HPLC system (Gilson Medical Electronics, Middleton, WI, USA) with two pumps (305 primary pump, 307 secondary pump) and a mixer (811C dynamic mixer), and a Shimadzu HPLC system (Shimadzu Co., Ltd., Kyoto, Japan) with a pump (LC-20AT), UV/vis detector (SPD-20A), and a system controller (CBM-20A) using a reversed-phase column (Watchers 120 ODS-BP, S-10 μm, 150 × 10 mm, Isu Industry Corp., Seoul, Republic of Korea). The resin for open-column chromatography used for silica gel 60 had a pore size of 6 nm, particle size of 63–200 µm (Product No. 1.07734, Merck, Darmstadt, Germany), and included Sephadex™ LH-20 (bead size 25–100 µm; GE Healthcare Bio-Sciences AB, Uppsala, Sweden). The gel-coated plate used in the thin-layer chromatography (TLC) detection assay was silica gel 60 F254 Art 5175 (GF254, 0.25 mm, Merck, Darmstadt, Germany), and spots were observed with UV spectrum and anisaldehyde-sulfuric acid reagent.

### 3.2. Plant Material

The root parts of *N. peltata* were collected in the Hantaek Botanical Garden Foundation, Yongin-si, Gyeonggi-do, Republic of Korea, in June 2021. The plants were authenticated by Dr. Jung Hwa Kang. A voucher specimen (PNU-0040) was deposited in the Medicinal Herb Garden, Pusan National University.

### 3.3. Extraction and Isolation

The dried and powdered root parts of *N. peltata* (2.8 kg) were ultrasonically extracted twice with MeOH (28 L, 90 min each) at room temperature. The solvent was concentrated under reduced pressure at 45 °C and immediately freeze-dried to obtain the crude *N. peltata* extract (427.02 g). The crude extract obtained was suspended in H_2_O (2 L) and then partitioned with *n*-hexane (Hx), ethyl acetate (EtOAc), and *n*-butanol (BuOH) to obtain three fractions, namely Hx (80.3 g), EtOAc (24.02 g), and BuOH (108.01 g) soluble fractions. The EtOAc soluble fraction was subjected to normal-phase silica column chromatography using a gradient mobile phase (EtOAc:MeOH from 10:1 to 100% MeOH) to obtain 7 subfractions (E1~E7). E2 (1.03 g) and was separated into 9 subfractions (E2-1~E2-9) by normal-phase silica column chromatography using a gradient mobile phase (chloroform (CHCl_3_):MeOH from 20:1 to 100% MeOH). E2-6 (52.53 mg) was separated into 4 subfractions (E2-6-1~E2-6-4) by normal-phase silica column chromatography using a gradient mobile phase (dichloromethane (CH_2_Cl_2_):MeOH from 10:1 to 100% MeOH). The E2-6-3 (15.34 mg) was subjected to a Shimadzu preparative-HPLC system (UV wavelength at 250 and 330 nm; flow rate 2 mL/min) using an isocratic mobile phase (0.1% formic acid + acetonitrile (MeCN):0.1% formic acid + H_2_O = 27:73) to obtain compounds **5** (3.7 mg; yield: 0.00087%; *t_R_* = 23 min) and **2** (0.7 mg; yield: 0.00016%; *t_R_ =* 44 min). E3 (1.10 g) was separated into 6 subfractions (E3-1~E3-6) by Sephadex LH-20 using 100% MeOH. E3-2 (357.02 mg) was separated into 5 subfractions (E3-2-1~E3-2-5) by normal-phase silica column chromatography using a gradient mobile phase (EtOAc:MeOH from 20:1 to 100% MeOH). E3-2-3 (137.91 mg) was separated into 3 subfractions (E3-2-3-1~E3-2-3-3) by Sephadex LH-20 using 100% MeOH. E3-2-3-2 (67.81 mg) was subjected to a Shimadzu preparative-HPLC system (UV wavelength at 250 and 330 nm; flow rate 2 mL/min) using an isocratic mobile phase (0.1% formic acid + MeCN:0.1% formic acid + H_2_O = 25:75) to obtain compound **6** (2.7 mg, yield: 0.00063%, *t_R_* = 78 min). E6 (5.98 g) was separated into 4 subfractions (E6-1~E6-4) by Gilson preparative-HPLC system (UV wavelength at 250 nm; flow rate 2 mL/min) using a gradient mobile phase (MeOH:H_2_O from 10:90 to 100% MeOH). E6-3 (397.12 mg) was separated into 7 subfractions (E6-3-1~E6-3-7) by Gilson preparative-HPLC system (UV wavelength at 250 nm; flow rate 2 mL/min) using an isocratic mobile phase (0.1% formic acid + MeCN:0.1% formic acid + H_2_O = 21:79). E6-3-7 (147.42 mg) was separated into 4 subfractions (E6-3-7-1~E6-3-7-4) by Shimadzu preparative-HPLC system (UV wavelength at 250 and 330 nm; flow rate 2 mL/min) using an isocratic mobile phase (0.1% formic acid + MeCN:0.1% formic acid + H_2_O = 30:70). E6-3-7-1 (25.02 mg) was subjected to a Shimadzu preparative-HPLC system (UV wavelength at 250 and 330 nm; flow rate 2 mL/min) using an isocratic mobile phase (0.1% formic acid + MeCN:0.1% formic acid + H_2_O = 22:78) to obtain compound **4** (3.4 mg; yield: 0.00080%; *t_R_* = 31 min). The BuOH soluble fraction was subjected to normal-phase silica column chromatography using a gradient mobile phase (EtOAc:MeOH from 20:1 to 100% MeOH) to obtain 5 subfractions (B1~B5). B4 (28.71 g) was separated into 5 subfractions (B4-1~B4-5) by normal-phase silica column chromatography using a gradient mobile phase (Hx:EtOAc from 10:1 to 100% EtOAc, to 100% MeOH, and to 100% H_2_O). B4-5 (193.41 g) was separated into 7 subfractions (B4-5-1~B4-5-7) by normal-phase silica column chromatography using a gradient mobile phase (CHCl_3_:MeOH from 20:1 to 100% MeOH). B4-5-3 (98.71 mg) was separated into 3 subfractions (B4-5-3-1~B4-5-3-3) by Sephadex LH-20 using 100% MeOH. B4-5-3-2 (17.51 mg) was subjected to a Shimadzu preparative-HPLC system (UV wavelength at 250 and 330 nm; flow rate 2 mL/min) using an isocratic mobile phase (0.1% formic acid + MeCN:0.1% formic acid + H_2_O = 20:80) to obtain compound **1** (2.4 mg; yield: 0.00056%; *t_R_* = 11 min). B5 (29.33 g) was separated into 7 subfractions (B5-1~B5-7) by normal-phase silica column chromatography using a gradient mobile phase (EtOAc:MeOH from 20:1 to 100% MeOH). B5-7 (19.96 g) was separated into 7 subfractions (B5-7-1~B5-7-7) by normal phase silica column chromatography using a gradient mobile phase (EtOAc:MeOH from 10:1 to 100% MeOH). B5-7-7 (5.20 g) was separated into 5 subfractions (B5-7-7-1~B5-7-7-5) by a Shimadzu preparative-HPLC system (UV wavelength at 250 and 330 nm; flow rate 2 mL/min) using an isocratic mobile phase (0.1% formic acid + MeCN:0.1% formic acid + H_2_O = 18:82). B5-7-7-5 (1.20 g) was separated into 3 subfractions (B5-7-7-5-1~B5-7-7-5-3) by a Gilson preparative-HPLC system (UV wavelength at 250 nm; flow rate 2 mL/min) using a gradient mobile phase (MeOH:H_2_O from 10:90 to 100% MeOH). The B5-7-7-5-1 (165.12 mg) was subjected to a Shimadzu preparative-HPLC system (UV wavelength at 250 and 330 nm; flow rate 2 mL/min) using an isocratic mobile phase (0.1% formic acid + MeCN:0.1% formic acid + H_2_O = 18:82) to obtain compound **3** (0.6 mg, yield: 0.00014%, *t_R_* = 54 min).

#### 3.3.1. Peltatamarin A (**5**)

Pale brown solid; [*α*]$\begin{array}{c} 25\\ D \end{array}$ + 16.1 (*c* 0.02, MeOH); UV (MeOH) *λ*_max_ 294, 327 nm (Figure S11); IR (KBr) *ν*_max_ 3385, 2980, 2840, 1705, 1510, 1330, 1013 cm^−1^; ^1^H (400 MHz, DMSO-*d*_6_) and ^13^C (100 MHz, DMSO-*d*_6_) NMR data, see Table 1; HR-ESI-MS (positive-ion-mode) *m*/*z* 539.1138 [M + Na]^+^ (calcd. for C_25_H_25_O_12_, 539.1165).

#### 3.3.2. Peltatamarin B (**6**)

Pale brown solid; [*α*]$\begin{array}{c} 25\\ D \end{array}$ + 16.0 (*c* 0.02, MeOH); UV (MeOH) *λ*_max_ 293, 325 nm (Figure S18); IR (KBr) *ν*_max_ 3380, 2978, 2835, 1702, 1513, 1327, 1015 cm^−1^; ^1^H (400 MHz, DMSO-*d*_6_) and ^13^C (125 MHz, DMSO-*d*_6_) NMR data, see Table 1; HR-ESI-MS (positive-ion-mode) *m*/*z* 553.1324 [M + Na]^+^ (calcd. for C_26_H_26_O_12_Na, 530.1322).

### 3.4. Acid Hydrolysis and Absolute Configuration Determination of Sugar Moieties

The absolute configuration of the sugar moieties was determined using an HPLC-UV-based method [28]. Compounds **5** (1.5 mg) and **6** (1.5 mg) were hydrolyzed in the presence of 1N HCl at 80 °C for 2 h, and EtOAc was used for the extraction. The aqueous layer was neutralized with repeated evaporation under a vacuum evaporator and dissolved in anhydrous pyridine (0.5 mL) with the addition of L-cysteine methyl ester hydrochloride (1.0 mg). After the reaction mixture was heated at 60 °C for 1 h, *o*-tolylisothiocyanate (50 μL) was added and the mixture was kept at 60 °C for 1 h. The reaction product was evaporated under a vacuum evaporator and dissolved in MeOH. Next, the dissolved reaction product was directly analyzed by a LC/MS (MeOH/H_2_O, 1:9 → 7:3 gradient system (0–20 min), 100% MeOH (21–31 min), 0% MeOH (32–42 min); flow rate of 0.3 mL/min) using an analytical Kinetex C_18_ 100 Å column (100 × 2.1 mm i.d., 5 μm). The sugar moiety of compounds **5** and **6** was identified as *β*-glucopyranoside, based on the comparison with the standard using LC/MS analysis.

### 3.5. Cell Culture

Human keratinocytes (HaCaT) were purchased from CLS Cell Lines Service GmbH (Eppelheim, Baden-Württemberg, Germany) and cultured as single layers at 37 °C in a 5% CO_2_ incubator (Forma Direct Heat CO_2_ Incubator, Thermo Fisher Scientific, Madison, WI, USA) in Dulbecco’s Modified Eagle’s Medium (DMEM; Gibco, Grand Island, NY, USA) supplemented with 10% fetal bovine serum (FBS; Gibco, Grand Island, NY, USA), 100 U/mL penicillin, and 100 μg/mL streptomycin (Gibco, Grand Island, NY, USA).

### 3.6. Wound Healing Assay

The wound healing assay was performed as previously described with some modification [31]. HaCaT cells were seeded in 24-well plates at a density of 2 × 10^5^ cells per well and allowed to attach for one day. Scratches were then created in each well using a 200 μL pipette tip. After washing with PBS, cells were treated with 2-bromo-palmitate (2BP) or lysophosphatidic acid (LPA) as negative and positive controls, respectively, at 10 μM or with compounds **1**~**6** at 10 μM. In addition, samples were cultured in a growth medium containing 1% FBS and 1% penicillin–streptomycin for 48 h to minimize the effects of growth factors. Wound images were captured immediately after wounding and after 24 or 48 h of culture using a microscope camera HK5.1 CMOS (Koptic, Yongin-si, Republic of Korea). Captured images were quantified using Image J software 1.52a (National Institutes of Health, Bethesda, MD, USA).

### 3.7. MTT Assay

HaCaT cells were seeded in 96-well plates at a density of 2 × 10^4^ cells per well in 100 μL. After washing the cells with DPBS (Dulbecco’s Phosphate-buffered Saline), cells were treated with 2BP or LPA as negative and positive controls, respectively, at 10 μM or with compound **5** at 1, 3, 10, 30, or 100 μM, and samples were cultured in DMEM containing 1% FBS for 24 h. After removing the supernatant, 100 μL of EZ-cytox assay reagent (Dogenbio, Seoul, Republic of Korea) was added, and the cells were incubated for 30 min. Subsequently, absorbance at 450 nm was measured using the Infinite M1000 microplate reader (Tecan, Mannedorf, Zürich, Switzerland) to assess cell viability.

### 3.8. Statistics Analysis

Data are expressed as the mean ± standard deviation (SD), and analysis was performed using GraphPad Prism software version 5.04 (GraphPad Software Inc., San Diego, CA, USA). The difference between the experimental groups was analyzed through the ANOVA test. Values of *p* < 0.05 were considered statistically significant.

## 4. Conclusions

In this study, two novel compounds, peltatamarins A (**5**) and B (**6**) and four known compounds were isolated and identified from the MeOH extract of *N. peltata* root. The structures of the two novel compounds were elucidated by spectroscopic analysis (1D and 2D NMR and HR-ESI-MS). Out of these isolated compounds, scopolin (**4**) and peltatamarin A (**5**) promoted wound repair. In addition, peltatamarin A (**5**) promoted cell migration dose-dependently in wound sites without influencing cell proliferation. These findings suggest that peltatamarin A (**5**) can have potential therapeutic use for treating wounds.

## Figures and Tables

**Table 1:** ^1^H and ^13^C NMR data for compounds **5** and **6** in DMSO-*d*_6_.

**Position**	**5**		**6**	
	***δ*_C_** **^b^**	***δ*_H_** **(*J* in Hz) ^a^**	***δ*_C_** **^c^**	***δ*_H_** **(*J* in Hz) ^a^**
1	160.9 C		160.8 C	
2				
3	113.9 CH	6.22 (d, 9.5)	113.8 CH	6.28 (d, 9.5)
4	144.5 CH	7.85 (d, 9.5)	144.4 CH	7.93 (d, 9.5)
5	113.2 CH	7.04 (s)	110.2 CH	7.29 (s)
6	144.0 C		146.2 C	
7	149.1 C		149.3 C	
8	103.6 CH	7.10 (s)	103.6 CH	7.19 (s)
9	148.3 C		148.8 C	
10	113.5 C		112.2 C	
1′	100.9 CH	5.02 (d, 7.5)	99.6 CH	5.20 (d, 7.5)
2′	73.5 CH	3.34 (m)	73.4 CH	3.34 (m)
3′	76.2 CH	3.33 (m)	76.9 CH	3.33 (m)
4′	70.4 CH	3.24 (m)	70.2 CH	3.23 (m)
5′	74.3 CH	3.77 (m)	74.1 CH	3.75 (m)
6′	63.8 CH_2_	4.41 (dd, 12.0, 2.0); 4.14 (dd, 12.0, 6.5)	63.8 CH_2_	4.40 (dd, 12.0, 2.0); 4.17 (dd, 12.0, 6.5)
1″	125.9 C		125.7 C	
2″	111.8 CH	7.21 (d, 2.0)	111.7 CH	7.23 (d, 2.0)
3″	148.4 C		148.5 C	
4″	149.9 C		150.0 C	
5″	116.0 CH	6.72 (d, 8.0)	116.2 CH	6.75 (d, 8.0)
6″	123.5 CH	7.03 (dd, 8.0, 2.0)	123.7 CH	7.01 (dd, 8.0, 2.0)
7″	145.9 CH	7.47 (d, 16.0)	145.7 CH	7.46 (d, 16.0)
8″	114.3 CH	6.39 (d, 16.0)	114.0 CH	6.39 (d, 16.0)
9″	167.1 C		166.5 C	
6-OCH_3_			56.5 CH_3_	3.81 (s)
3″-OCH_3_	56.1 CH_3_	3.76 (s)	56.0 CH_3_	3.79 (s)

^a^ Measured at 400 MHz in DMSO-*d*_6_. ^b^ Measured at 100 MHz in DMSO-*d*_6_. ^c^ Measured at 125 MHz in DMSO-*d*_6_.

## Data Availability

Data are contained within the article and Supplementary Materials.

## Supplementary Materials

The supporting information can be downloaded at: https://www.mdpi.com/article/10.3390/ijms25021268/s1.
