# Supplementary material for: Iridoid Glycosides and Coumarin Glycoside Derivatives from the Roots of Nymphoides peltata and Their In Vitro Wound Healing Properties
Source: Int J Mol Sci. 2024 Jan 19;25(2):1268. doi: 10.3390/ijms25021268 (PMC10816987; doi:10.3390/ijms25021268)

Article

Iridoid Glycosides and Coumarin Glycoside Derivatives from the Roots of *Nymphoides peltata* and Their In Vitro Wound Healing Properties

Tae-Young Kim [1,†], Bum Soo Lee [2,†], Beom-Geun Jo [1], Seong Pil Heo [3], Young Suk Jung [1], Su-Nam Kim [3,4], Ki Hyun Kim [2,*] and Min Hye Yang [1,*]

[1] Department of Pharmacy, College of Pharmacy and Research Institute for Drug Development,
Pusan National University, Busan 46241, Republic of Korea; taeyour@pusan.ac.kr (T.-Y.K.); bg_jo@pusan.ac.kr (B.-G.J.)
[2] School of Pharmacy, Sungkyunkwan University, Suwon 16419, Republic of Korea; kosboybs@skku.edu
[3] Natural Products Research Institute, Korea Institute of Science and Technology, Gangneung 25451, Republic of Korea; 123045@kist.re.kr (S.P.H.); snkim@kist.re.kr (S.-N.K.)
[4] Division of Bio-Medical Science and Technology, KIST School, University of Science and Technology,
Seoul 02792, Republic of Korea
* Correspondence: khkim83@skku.edu (K.H.K.); mhyang@pusan.ac.kr (M.H.Y.); Tel.: +82-51-510-2811 (M.H.Y.)
† These authors contributed equally to this work.

Citation: Kim, T.-Y.; Lee, B.S.; Jo, B.-G.; Heo, S.P.; Jung, Y.S.; Kim, S.-N.; Kim, K.H.; Yang, M.H. Iridoid Glycosides and Coumarin Glycoside Derivatives from the Roots of *Nymphoides peltata* and Their In Vitro Wound Healing Properties. *Int. J. Mol. Sci.* **2024**, *25*, x. https://doi.org/10.3390/xxxxx

Academic Editor: Arnaud Bianchi

Received: 16 December 2023
Revised: 12 January 2024
Accepted: 17 January 2024
Published: 19 January 2024

Copyright: © 2024 by the authors. Submitted for possible open access publication under the terms and conditions of the Creative Commons Attribution (CC BY) license (https://creativecommons.org/licenses/by/4.0/).

Contents of Supplementary material

Table S1. ^{1}H and ^{13}C NMR Spectrum Data of Compounds **1-4**

Figure S1. ^{1}H NMR spectrum of **1** in DMSO-d_6 (400 MHz)

Figure S2. ^{1}H NMR spectrum of **2** in DMSO-d_6 (400 MHz)

Figure S3. ^{1}H NMR spectrum of **3** in DMSO-d_6 (400 MHz)

Figure S4. ^{1}H NMR spectrum of **4** in DMSO-d_6 (500 MHz)

Figure S5. HR-ESI-MS data of **5**

Figure S6. ^{1}H NMR spectrum of **5** in DMSO-d_6 (400 MHz)

Figure S7. ^{13}C NMR spectrum of **5** in DMSO-d_6 (100 MHz)

Figure S8. ^{1}H-^{1}H COSY spectrum of **5** in DMSO-d_6

Figure S9. HSQC spectrum of **5** in DMSO-d_6

Figure S10. HMBC spectrum of **5** in DMSO-d_6

Figure S11. UV/PDA spectrum of **5**

Figure S12. HR-ESI-MS data of **6**

Figure S13. ^{1}H NMR spectrum of **6** in DMSO-d_6 (400 MHz)

Figure S14. ^{13}C NMR spectrum of **6** in DMSO-d_6 (125 MHz)

Figure S15. ^{1}H-^{1}H COSY spectrum of **6** in DMSO-d_6

Figure S16. HSQC spectrum of **6** in DMSO-d_6

Figure S17. HMBC spectrum of **6** in DMSO-d_6

Figure S18. UV/PDA spectrum of **6**

Figure S19. Wound healing area of compounds isolated from *N. peltate*

Table S1. ^{1}H and ^{13}C NMR spectrum data of compounds **1~4**

Position	Compound **1**		Compound **2**		Compound **3**		Compound **4**	
	δ_C, type [c]	δ_H (J in Hz) [a]	δ_C, type [d]	δ_H (J in Hz) [a]	δ_C, type [c]	δ_H (J in Hz) [a]	δ_C, type [c]	δ_H (J in Hz) [b]
1	96.7 CH	5.46 d (2.0)	95.7 CH	5.36 (d, 2.0)	96.3 CH	5.12 (dd, 9.0, 5.5)		
2							161.0 C	
3	153.7 CH	7.57 d (2.5)	146.9 CH	7.22 (d, 2.5)	151.0 CH	7.33 (d, 1.0)	113.8 CH	6.33 (d, 9.5)
4	103.6 C		107.2 C		113.0 C		144.7 CH	7.97 (d, 9.5)
5	23.9 CH_2	3.22 (m)	24.1 CH	2.94 (m)	31.9 CH	2.96 (m)	110.2 CH	7.30 (s)
6	28.3 CH_2	1.92 (m)	30.8 CH_2	2.10 (dt, 12.0, 4.0)<br>1.08 (m)	40.0 CH_2	2.16 (m),<br>1.68 (ddd, 13.7, 8.5, 4.0)	146.5 C	
7	92.3 CH	6.76 (td, 7.5, 1.5)	86.7 CH	4.93 (dd, 9.5, 4.0)	77.8 CH	5.12 (dd, 9.0, 5.5)	150.4 C	
8	132.5 CH	5.46 (m)	133.1 CH	5.43 (m)	40.0 CH	2.04 (m)	103.5 CH	7.16 (s)
9	41.4 CH	2.69 (m)	42.8 CH_2	2.62 (ddd, 8.0, 4.0, 2.0)	45.7 CH	1.89 (m)	149.4 C	
10	121.3 CH_2	5.27 (m)	120.7 CH_2	5.26 (dd, 17.0, 2.5),<br>5.19 (dd, 10.0, 2.5)	14.0 CH_3	0.96 (d, 7.0)	112.8 C	
11	163.3 C		160.9 C		168.5 C			
1′	99.1 CH	4.51 d (8.0)	98.8 CH	4.47 (d, 8.0)	99.2 CH	4.47 (d, 8.0)	100.1 CH	5.08 (d, 7.0)
2′	77.2 CH	3.00 (m)	77.1 CH	3.10 (m)	77.2 CH	3.10 (m)	77.6 CH	3.30 (m)
3′	71.9 CH	2.95 (m)	73.4 CH	3.10 (m)	73.7 CH	2.93 (m)	73.5 CH	3.30 (m)
4′	70.5 CH	3.15 (m)	70.4 CH	3.10 (m)	70.6 CH	2.96 (m)	70.1 CH	3.30 (m)
5′	73.6 CH	3.12 (m)	77.0 CH	3.10 (m)	76.7 CH	3.10 (m)	77.2 CH	3.30 (m)
6′	61.5 CH_2	3.65 (ddd, 12.0, 6.5, 2.0),<br>3.39 (m)	61.4 CH_2	3.64 (dd, 10.5, 5.0)	61.7 CH_2	3.64 (d, 12.0),<br>3.40 (overlap)	61.1 CH_2	3.44 (d, 8.0)
OCH_3							56.5 CH_3	3.82 (s)
1″	165.6 C		171.6 C		125.6 C			
2″	126.6 C		64.1 CH	3.78 (d, 7.5)	130.9 CH	7.53 (d, 9.0)		
3″	145.8 CH	6.51 (t, 2.4)	77.7 CH	3.94 (m)	116.2 CH	6.74 (d, 9.0)		
4″	21.9 CH	2.12 (ddp, 23.7, 15.0, 8.0)	19.6 CH_3	1.30 d (6.0)	160.3 C			
5″	40.7 CH	1.47 (ddd, 10.5, 6.0, 4.0)			116.2 CH	6.74 (d, 9.0)		
6″	71.9 CH				130.9 CH	7.53 (d, 9.0)		
7″	146.2 CH_2	5.83 (dd, 17.0, 10.5)			145.2 CH	6.36 (d, 16.0)		
8″	111.8 CH_2	5.12 (dd ,17.0, 2.0),<br>4.93 (m)			114.9 CH	7.53 (d, 16.0)		
9″	12.5 CH_3	1.73 (s)			166.8 C			

[a]Measured at 400 MHz in DMSO-d_6. [b] Measured at 500 MHz in DMSO-d_6. [c] Measured at 100 MHz in DMSO-d_6. [d] Measured at 125 MHz in DMSO-d_6.

Figure S1. ^{1}H NMR spectrum of **1** in DMSO-d_6 (400 MHz)

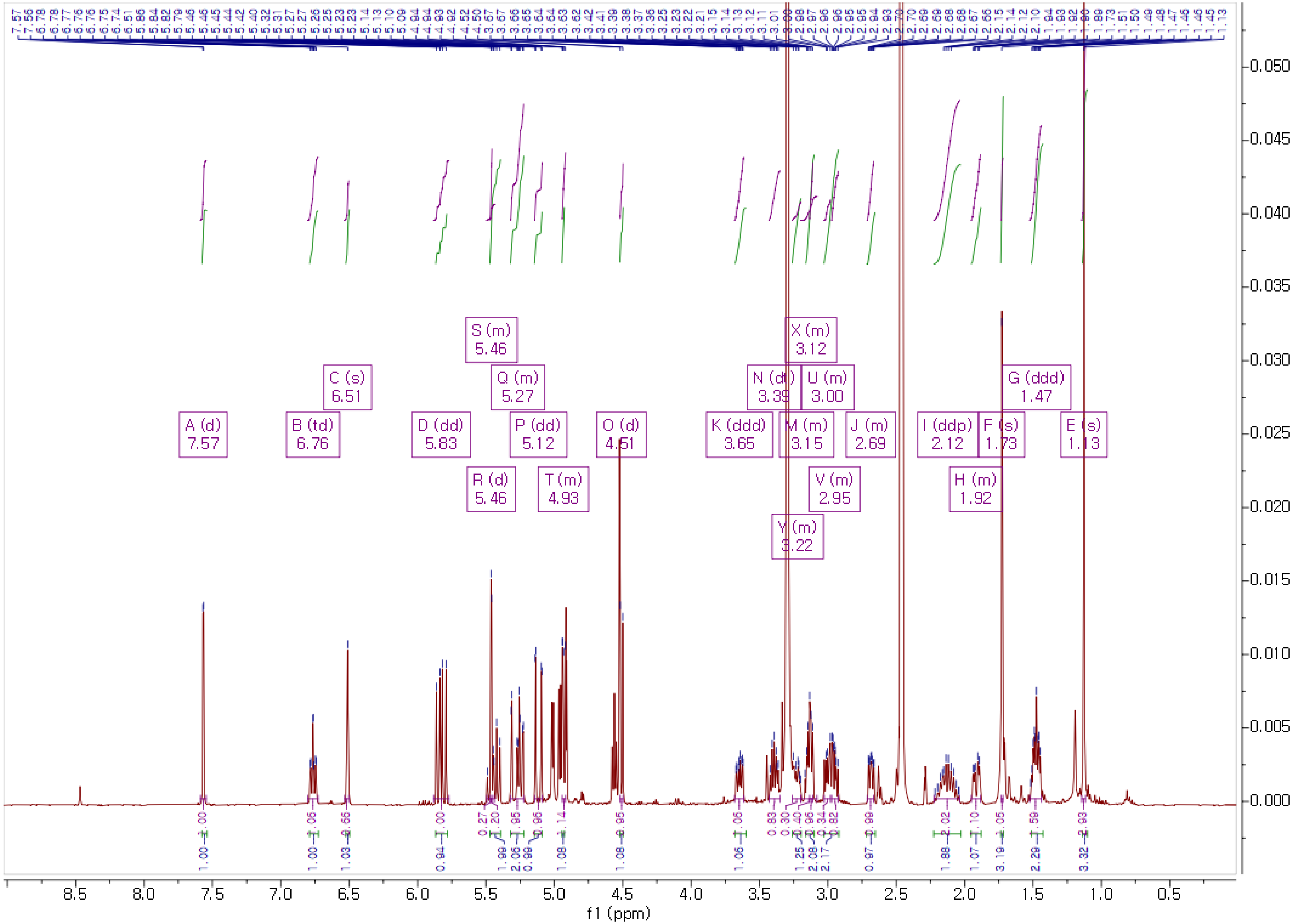

Figure S2. ^{1}H NMR spectrum of **2** in DMSO-d_6 (400 MHz)

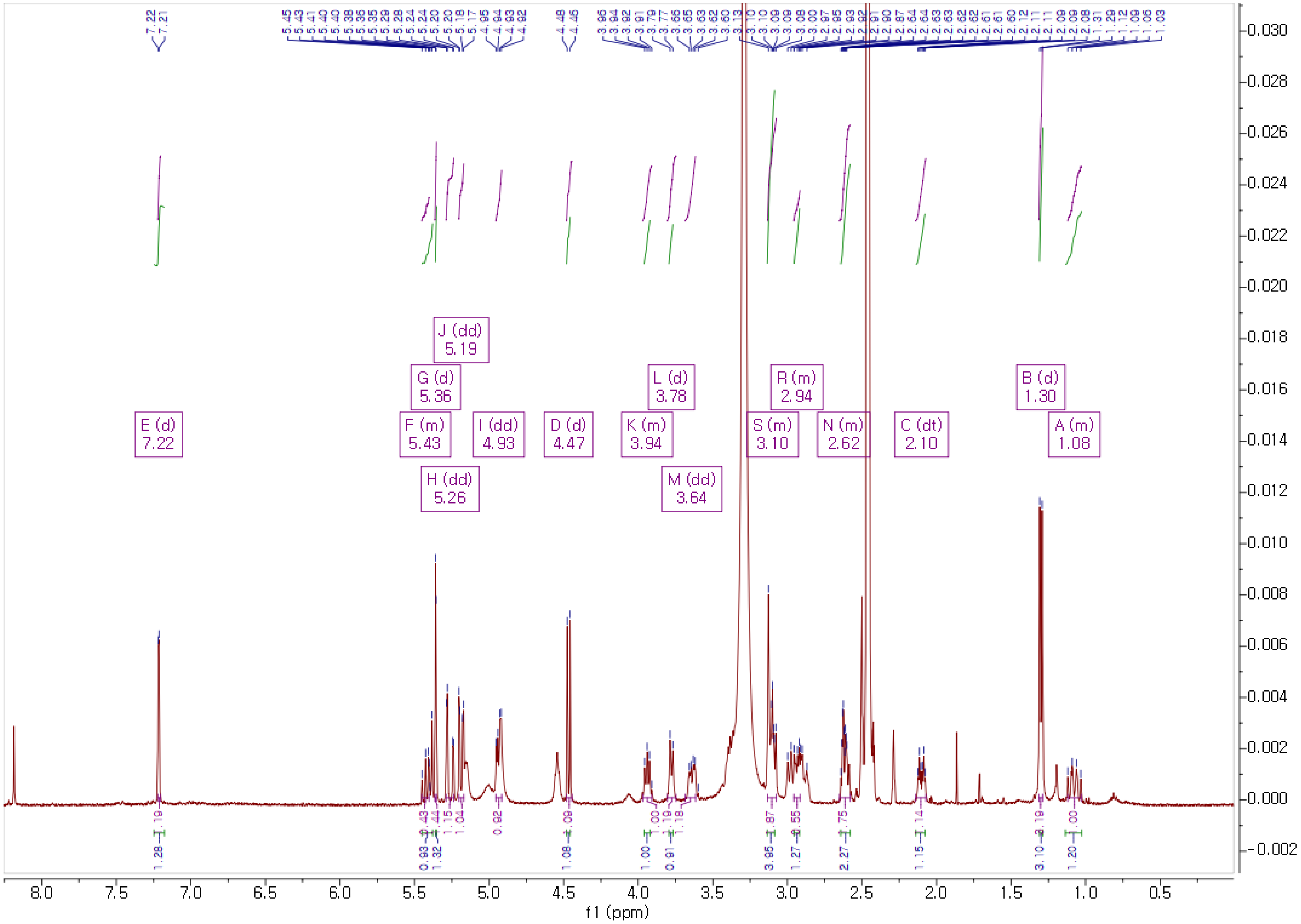

Figure S3. ^1H NMR spectrum of **3** in DMSO-d_6 (400 MHz)

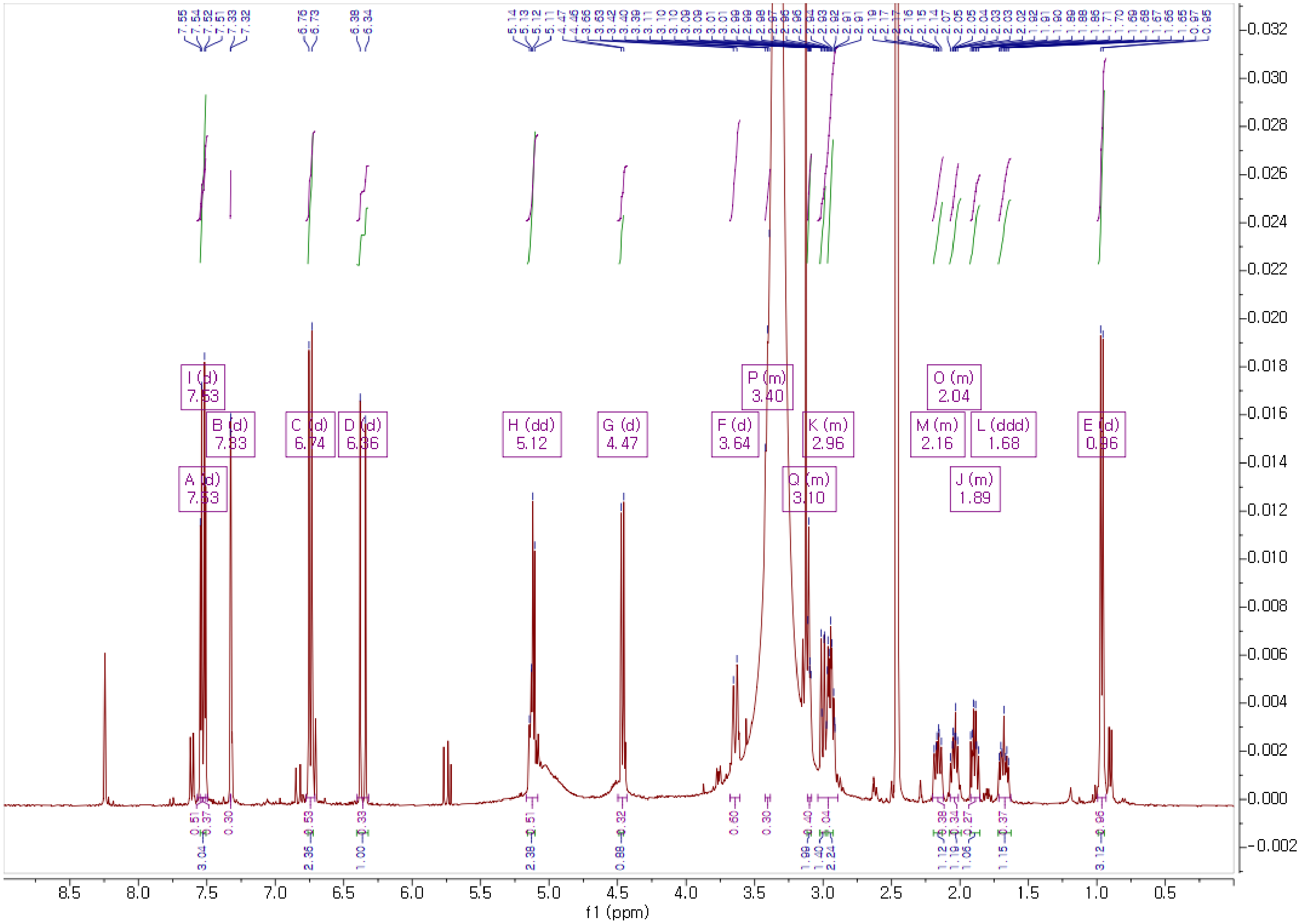

Figure S4. ^{1}H NMR spectrum of **4** in DMSO-d_6 (500 MHz)

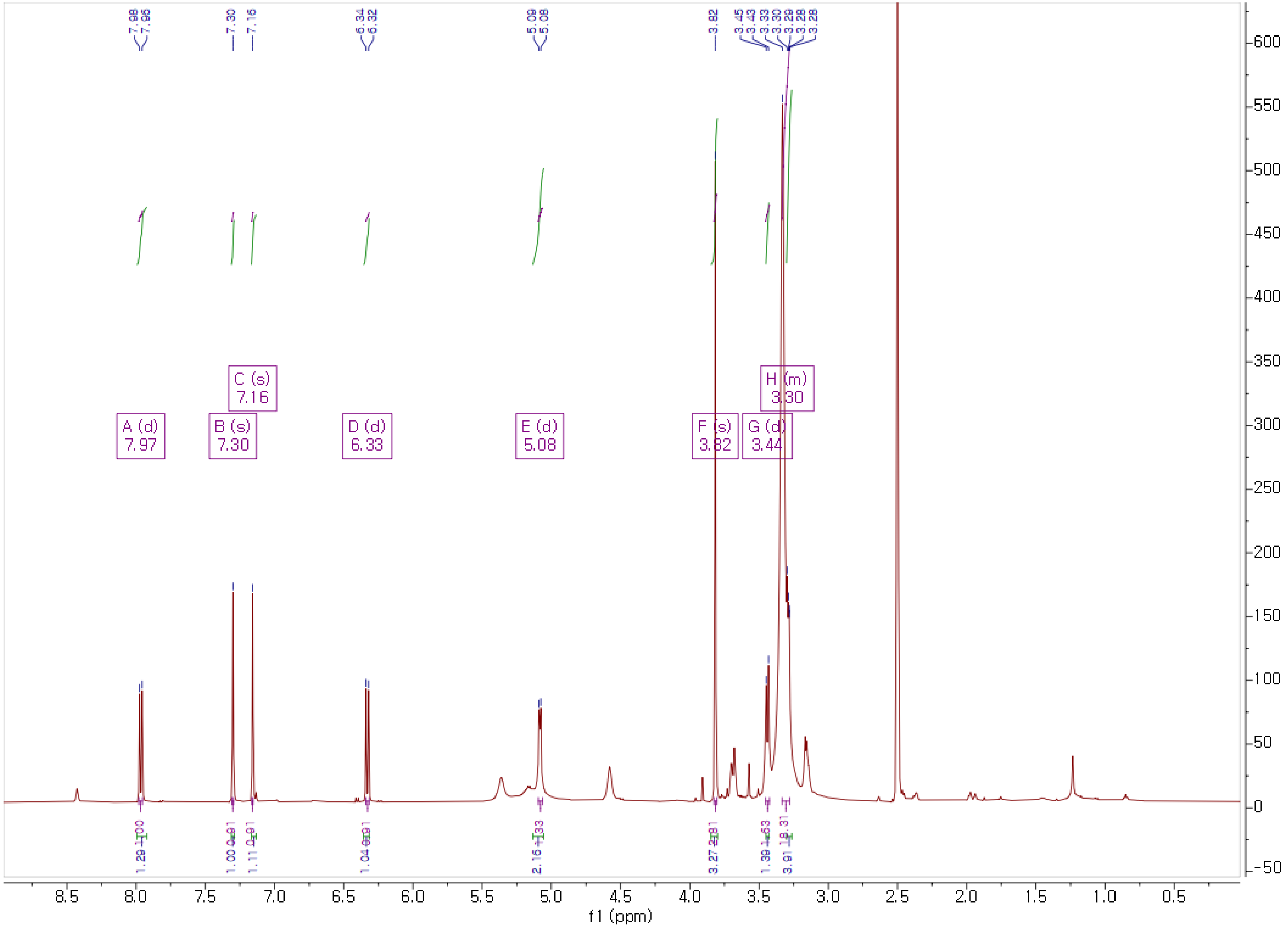

Figure S5. HR-ESI-MS data of **5**

Compound Identification Results: Cpd 1220: C25 H24 O12; 4.096

ID Techniques Applied

MFG

Best	Name	Formula	m/z	Mass	Species	Diff (ppm)	RT	Score
⦿		C25 H24 O12	539.1138	516.1251	(M+Na)+	-3.22	4.096	91.42
○		C32 H20 O7	517.1285 539.1138	516.1221	(M+H)+ (M+Na)+	2.4	4.096	77.64
○		C14 H28 O20	517.1285	516.1222	(M+H)+	9.31	4.096	47.09

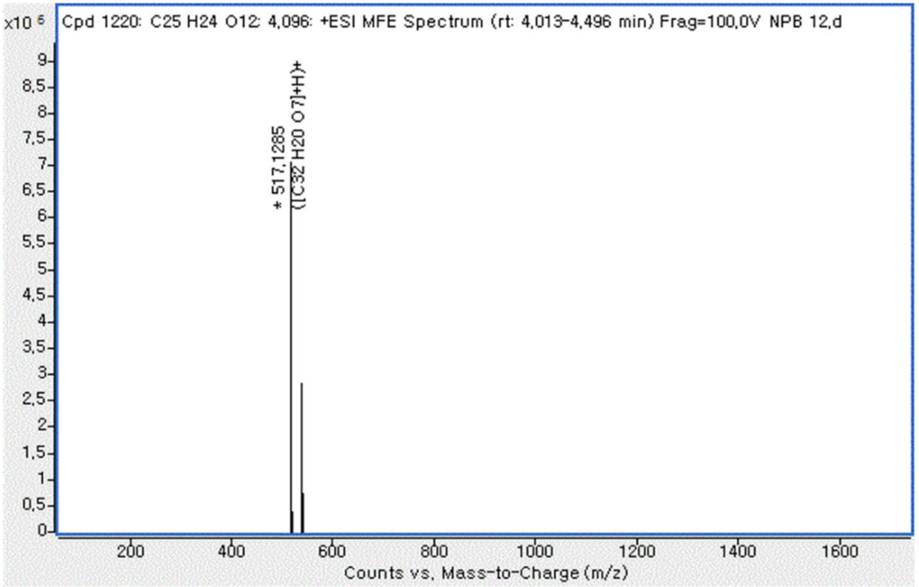

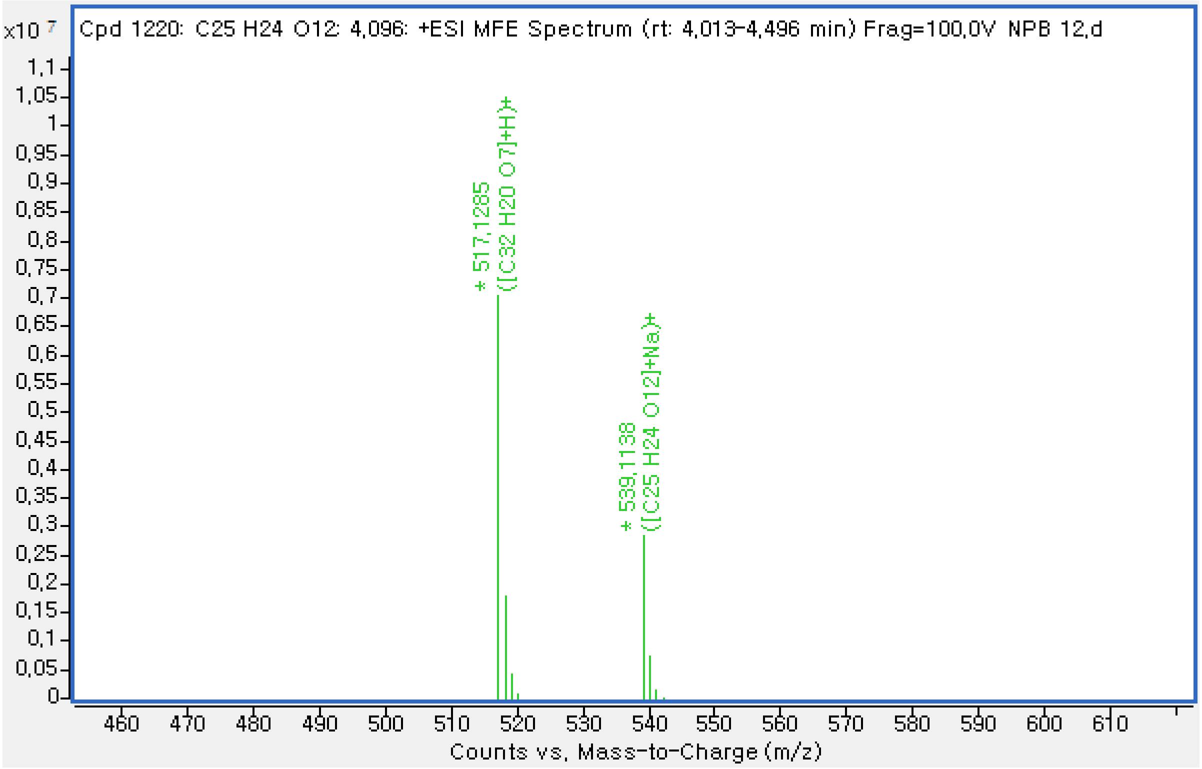

x10 7
Cpd 1220: C25 H24 O12: 4,096: +ESI MFE Spectrum (rt: 4,013-4,496 min) Frag=100,0V NPB 12,d
1,1
1,05
1
0,95
0,9
0,85
0,8
0,75
0,7
0,65
0,6
0,55
0,5
0,45
0,4
0,35
0,3
0,25
0,2
0,15
0,1
0,05
0
* 517,1285
([C32 H20 O7]+H)+
* 539,1138
([C25 H24 O12]+Na)+
460 470 480 490 500 510 520 530 540 550 560 570 580 590 600 610
Counts vs. Mass-to-Charge (m/z)

Figure S6. ^{1}H NMR spectrum of **5** in DMSO-d_6 (400 MHz)

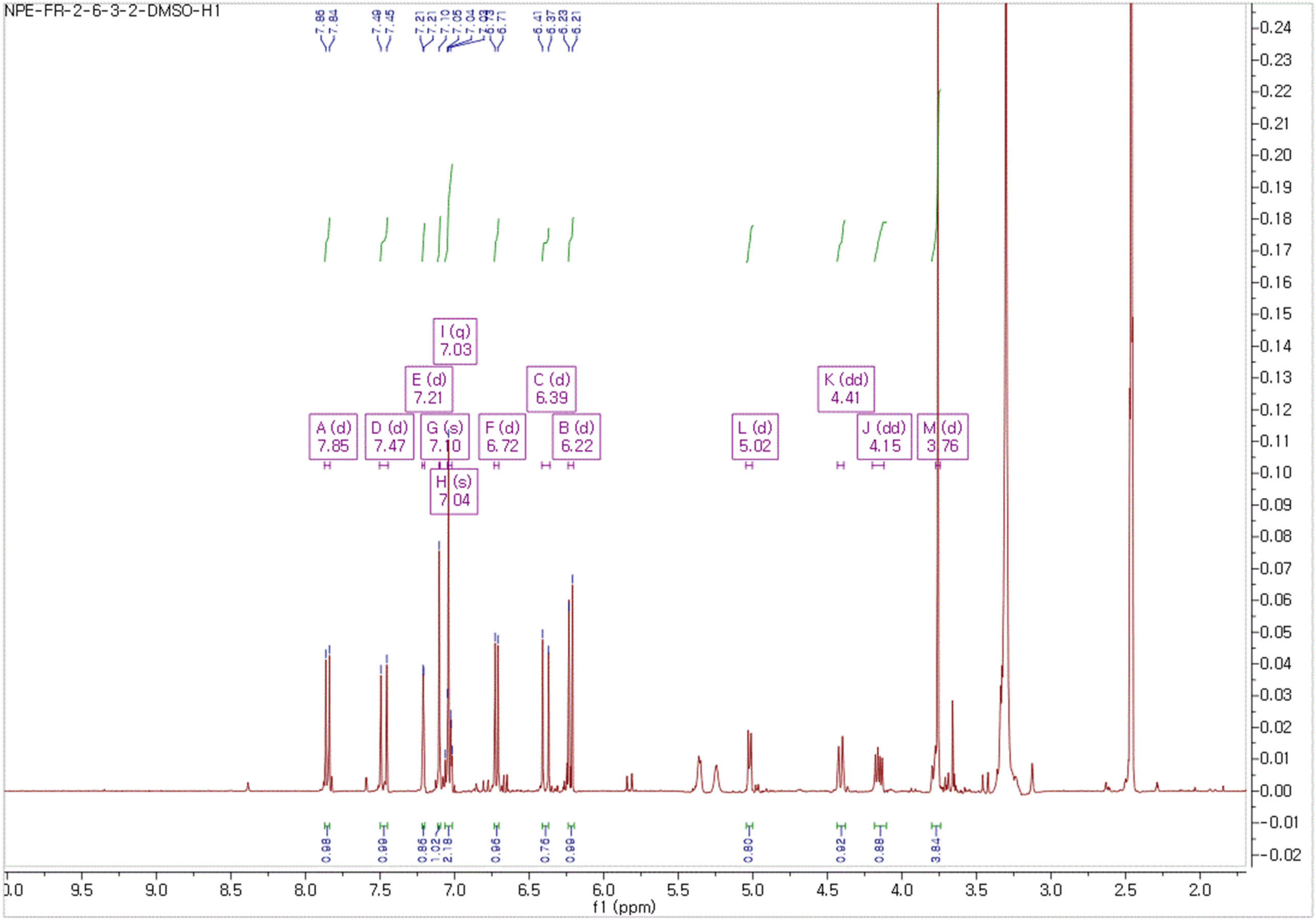

Figure S7. ^{13}C NMR spectrum of **5** in DMSO-d_6 (100 MHz)

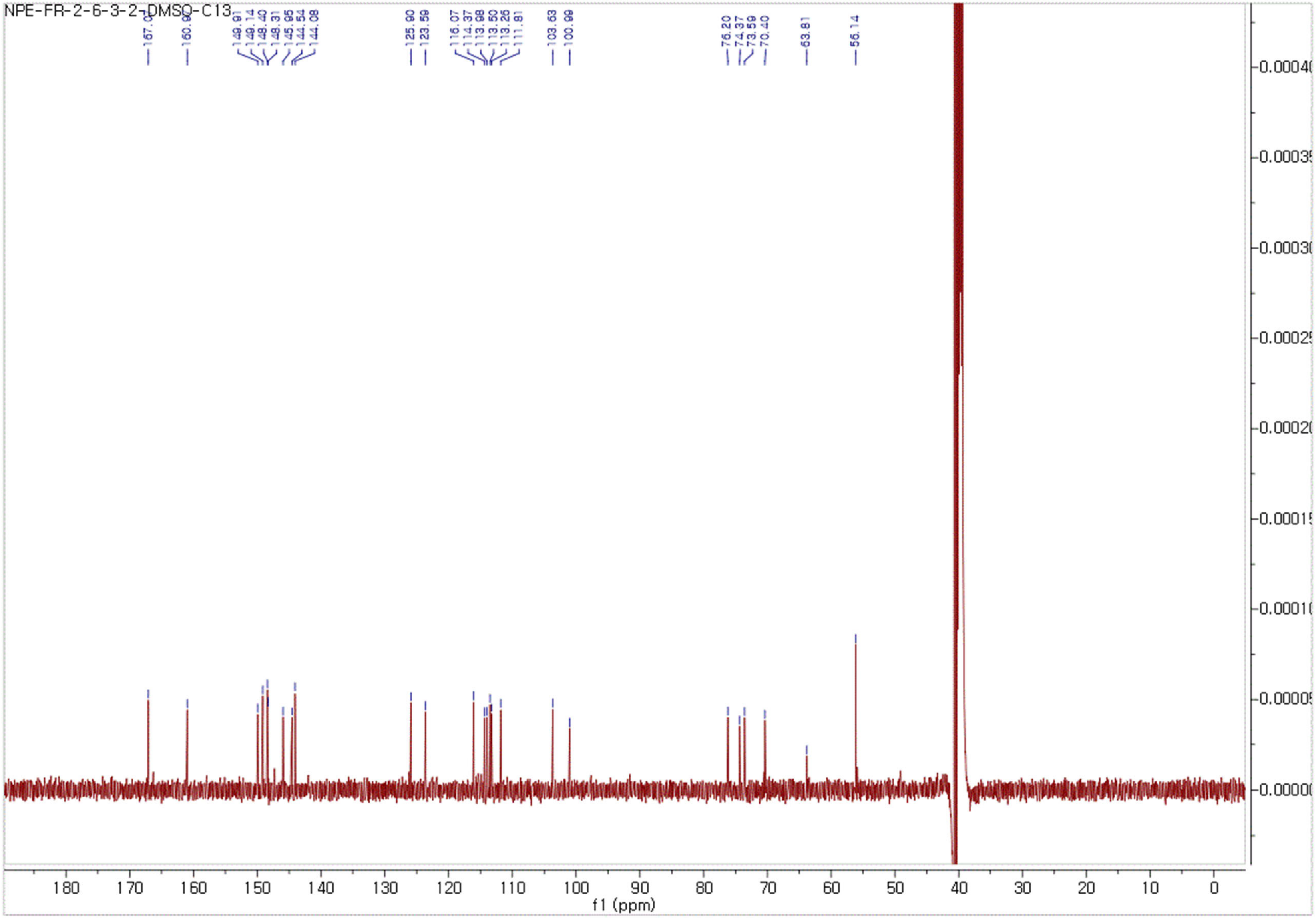

Figure S8. ^{1}H-^{1}H COSY spectrum of **5** in DMSO-d_6

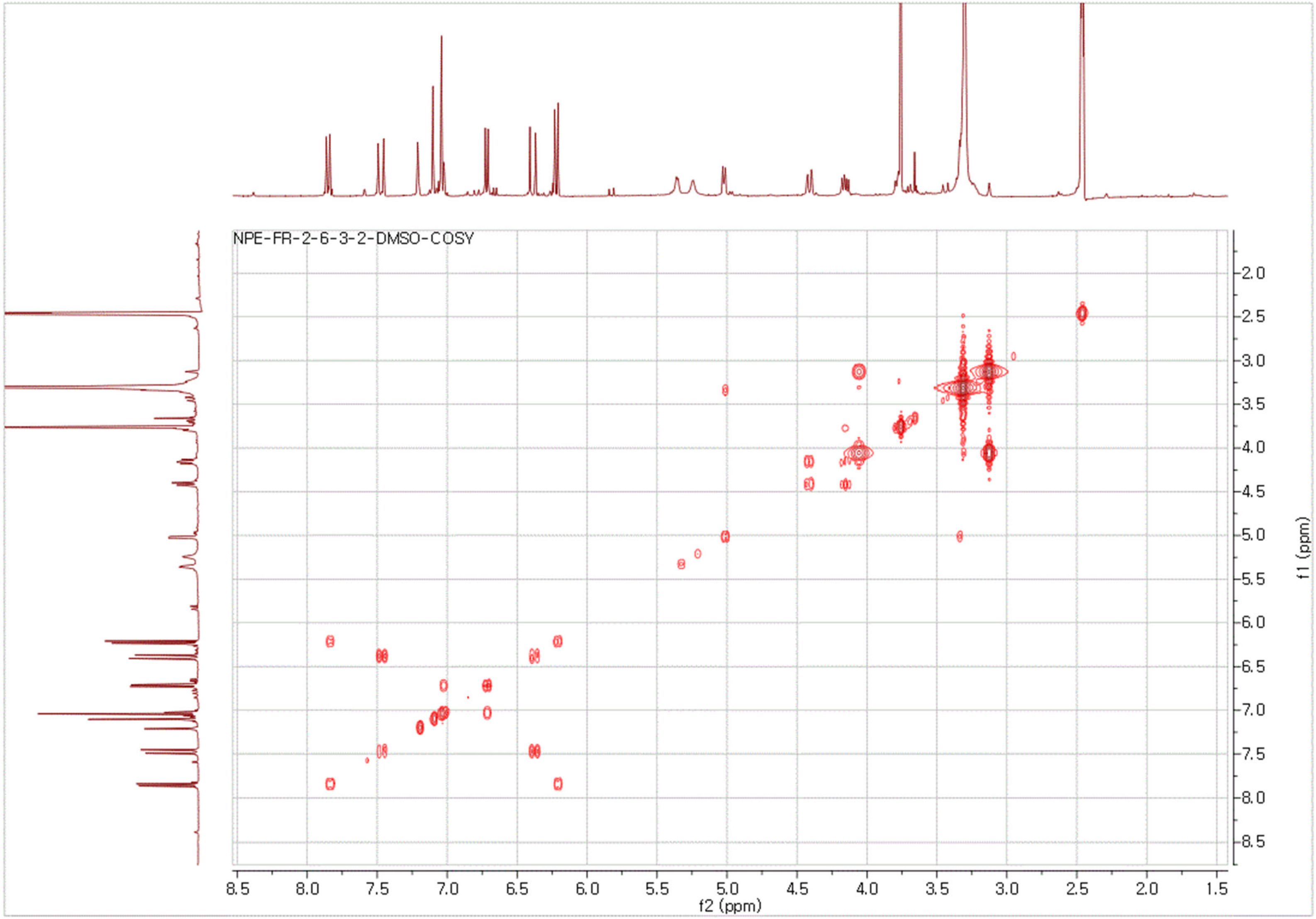

Figure S9. HSQC spectrum of **5** in DMSO-d_6

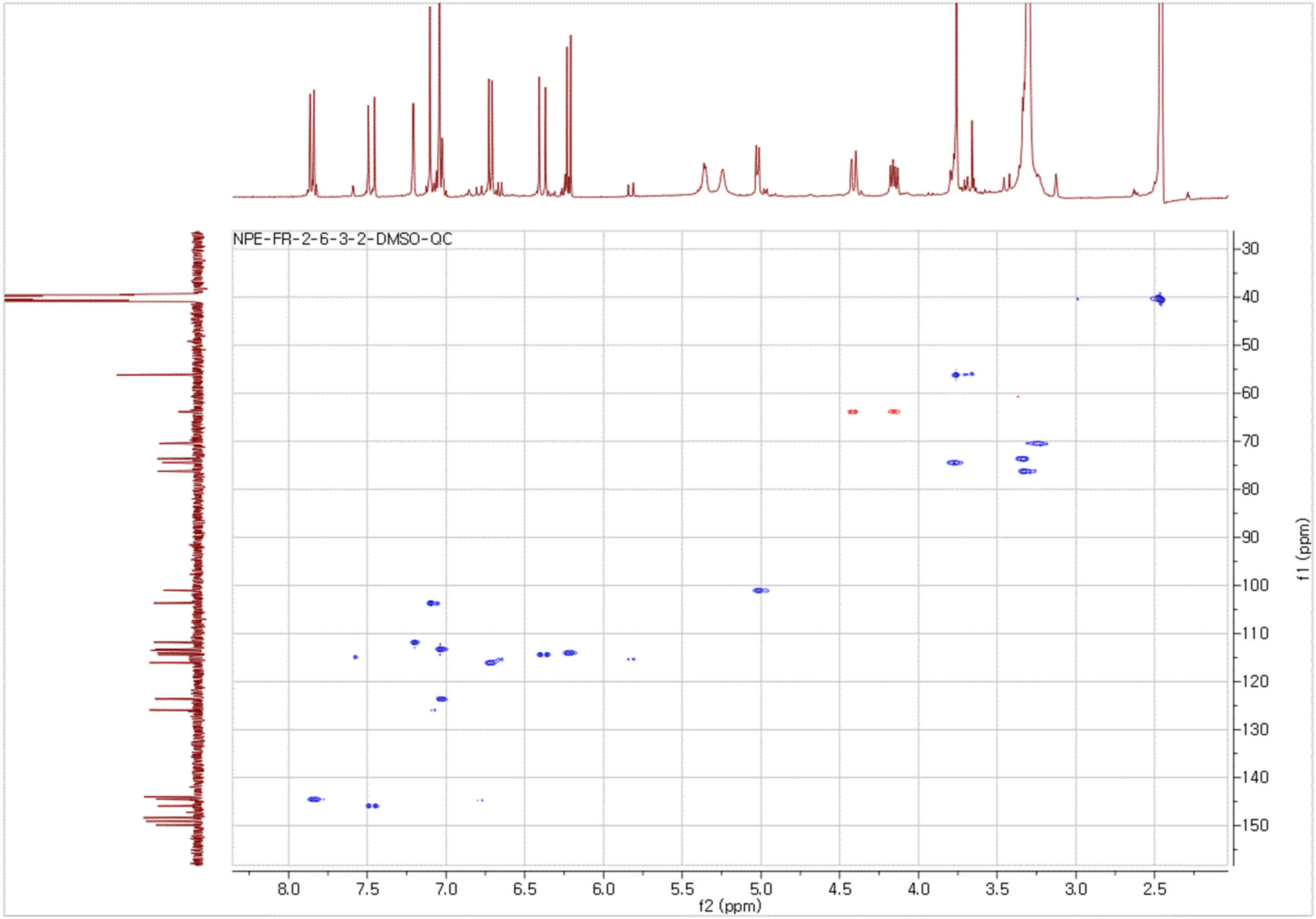

Figure S10. HMBC spectrum of **5** in DMSO-*d*6

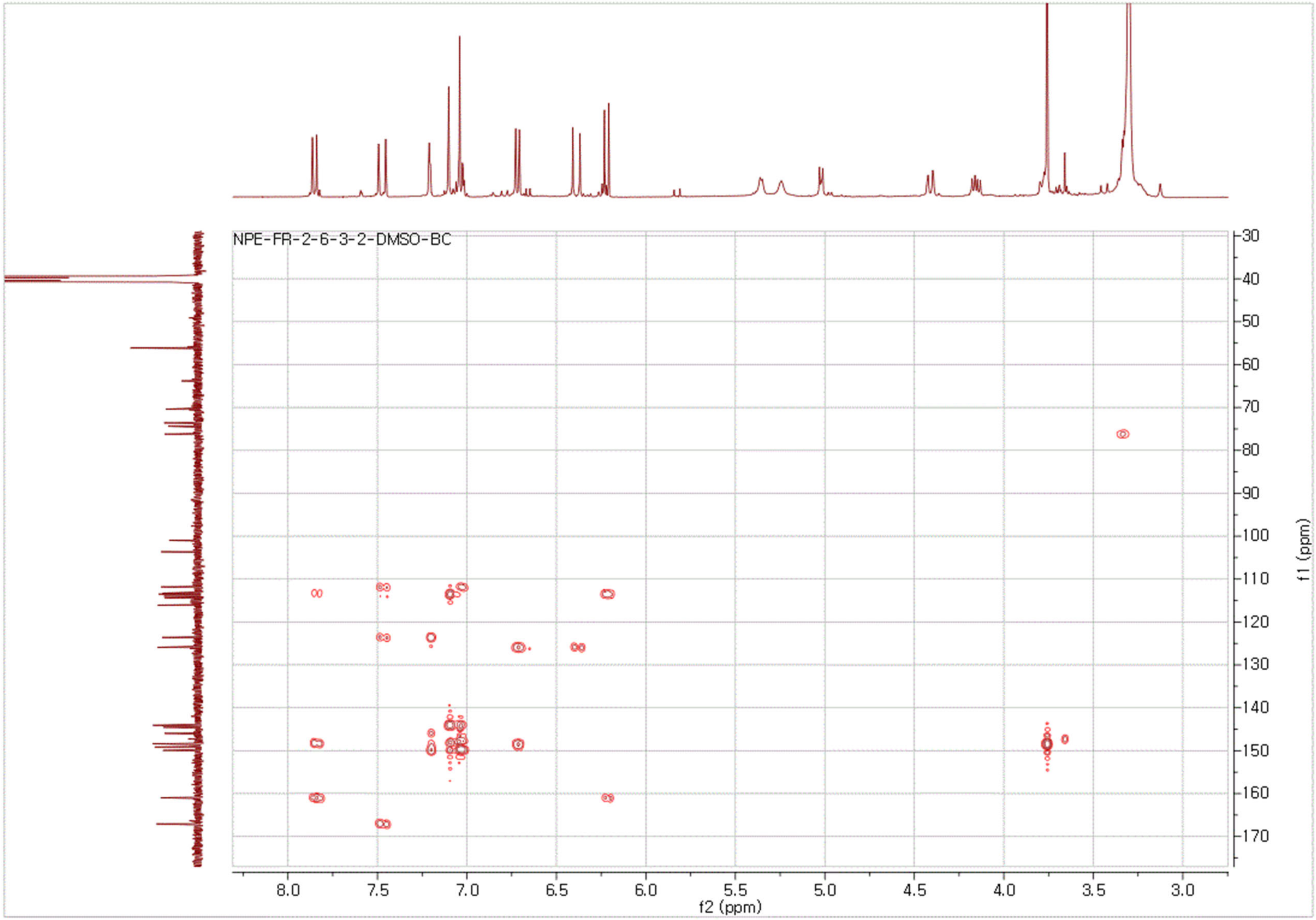

Figure S11. UV/PDA spectrum of **5**

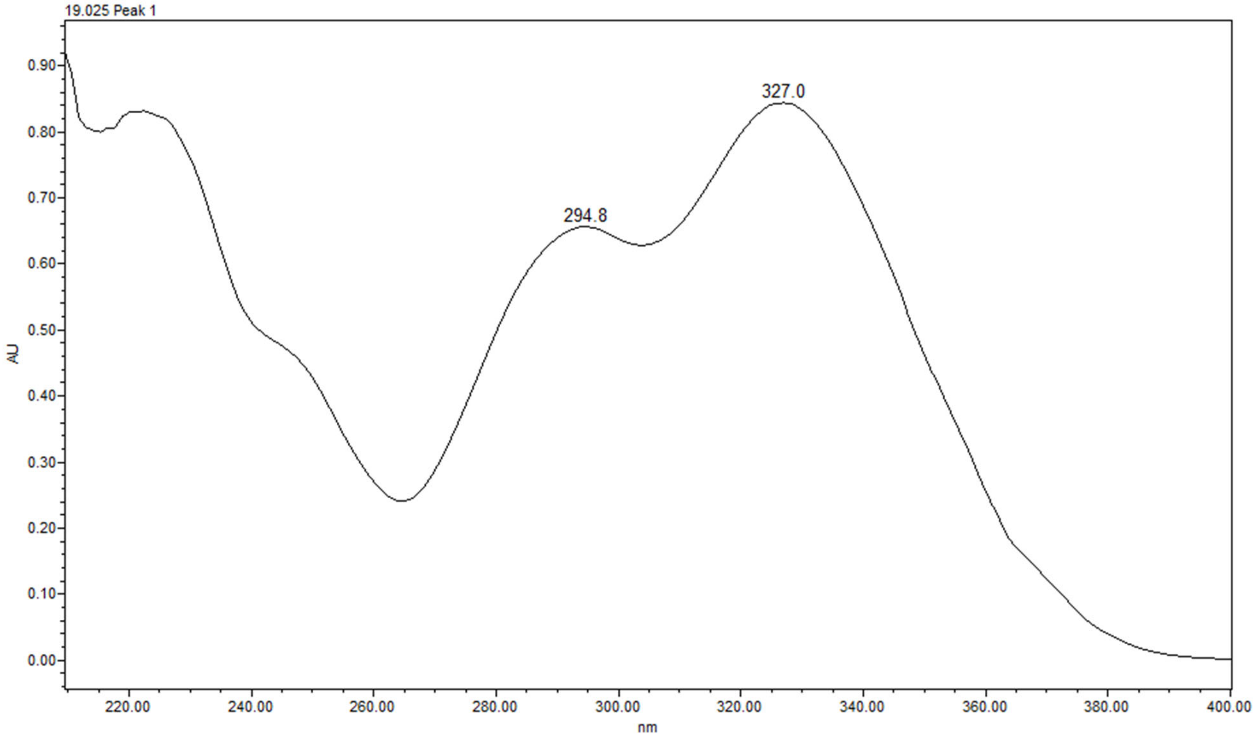

Figure S12. HR-ESI-MS data of **6**

Compound Identification Results: Cpd 833: C26 H26 O12; 4.214

ID Techniques Applied: MFG

Best	Name	Formula	m/z	Mass	Species	Diff (ppm)	RT	Score
⊙		C26 H26 O12	531.1504 548.1770 553.1324 1061.2918 1083.2751	530.142	(M+H)+ (M+NH4)+ (M+Na)+ (2M+H)+ (2M+Na)+	-0.8	4.214	99.37

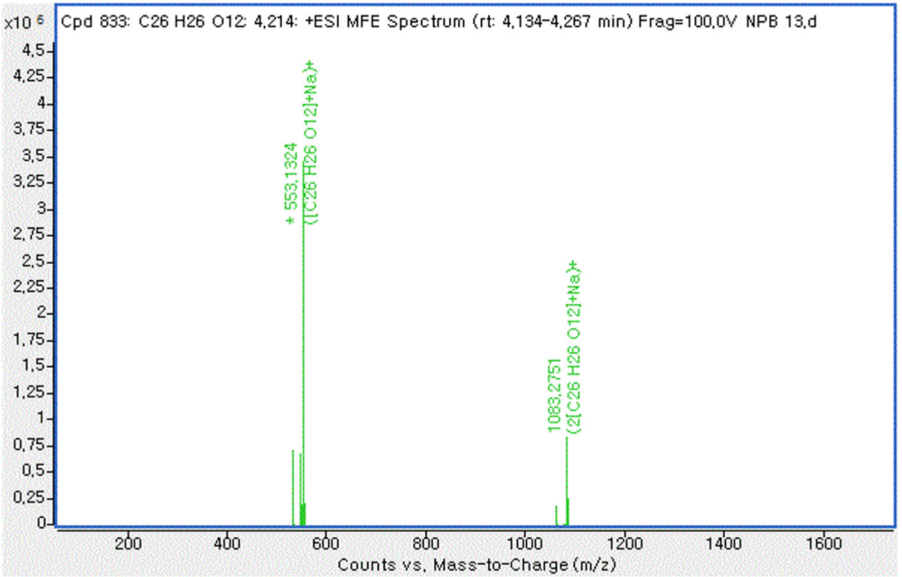

Figure S13. ^{1}H NMR spectrum of **6** in DMSO-d_6 (400 MHz)

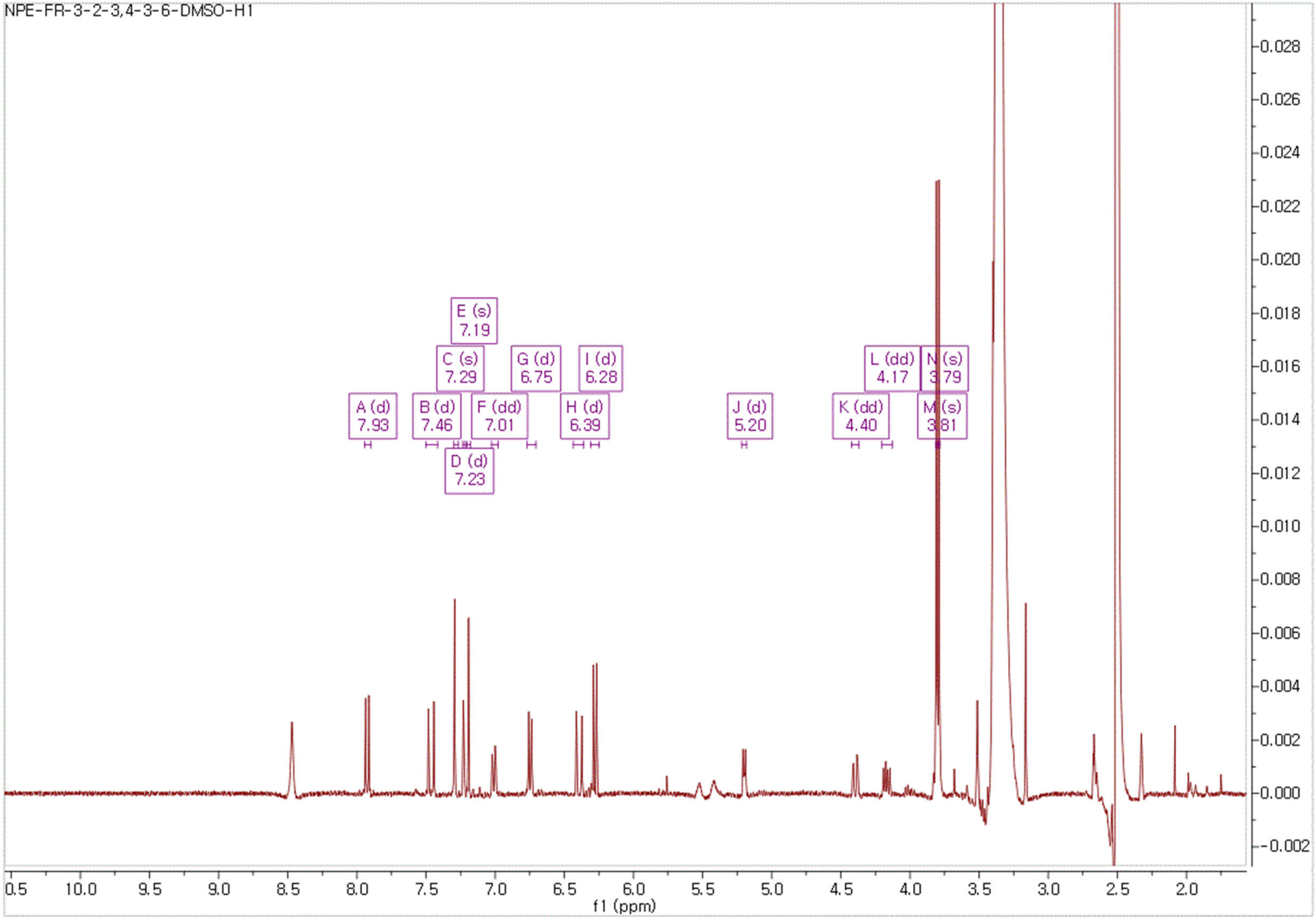

Figure S14. ^{13}C NMR spectrum of **6** in DMSO-d_6 (125 MHz)

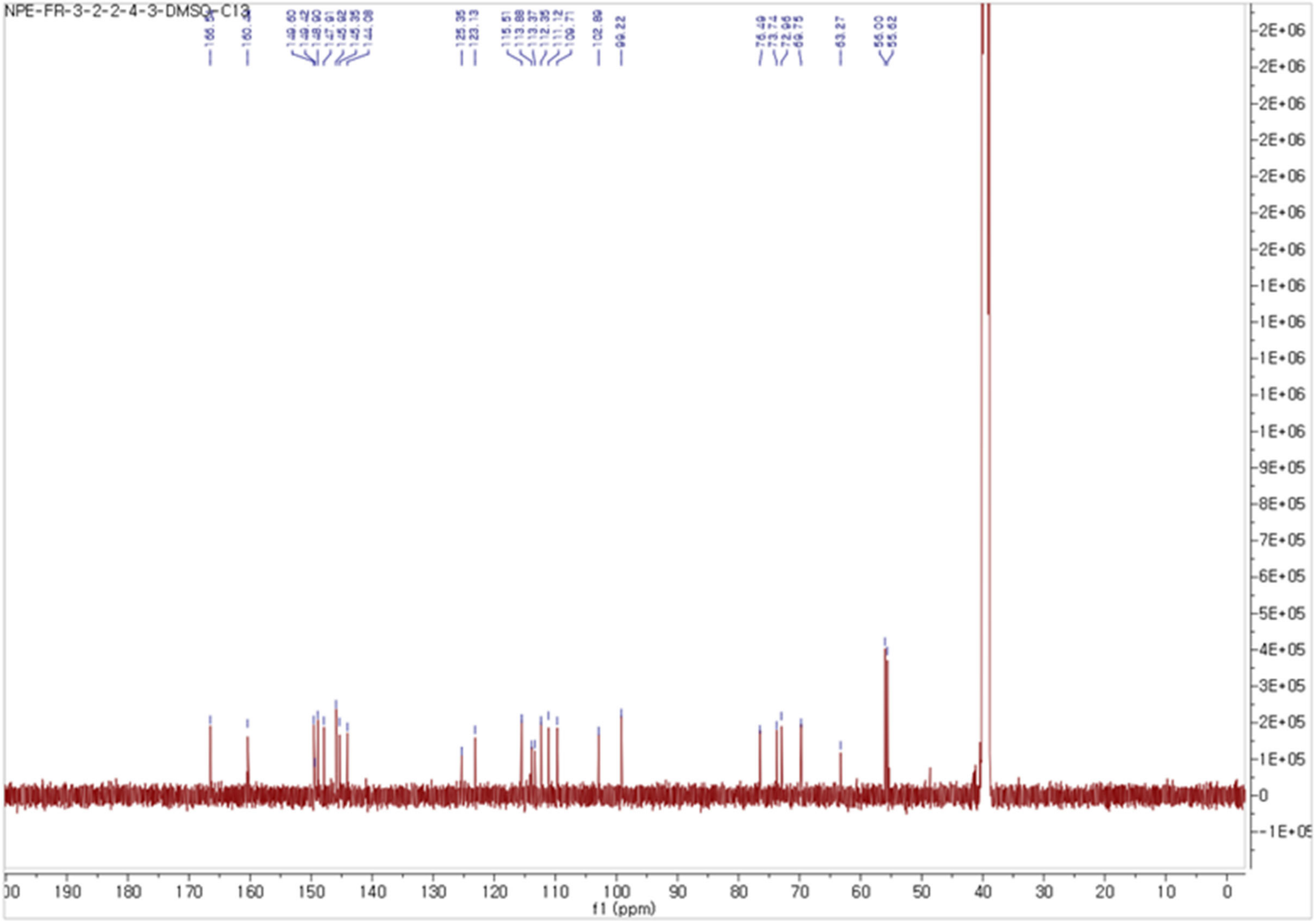

Figure S15. ^1H-^1H COSY spectrum of **6** in DMSO-d_6

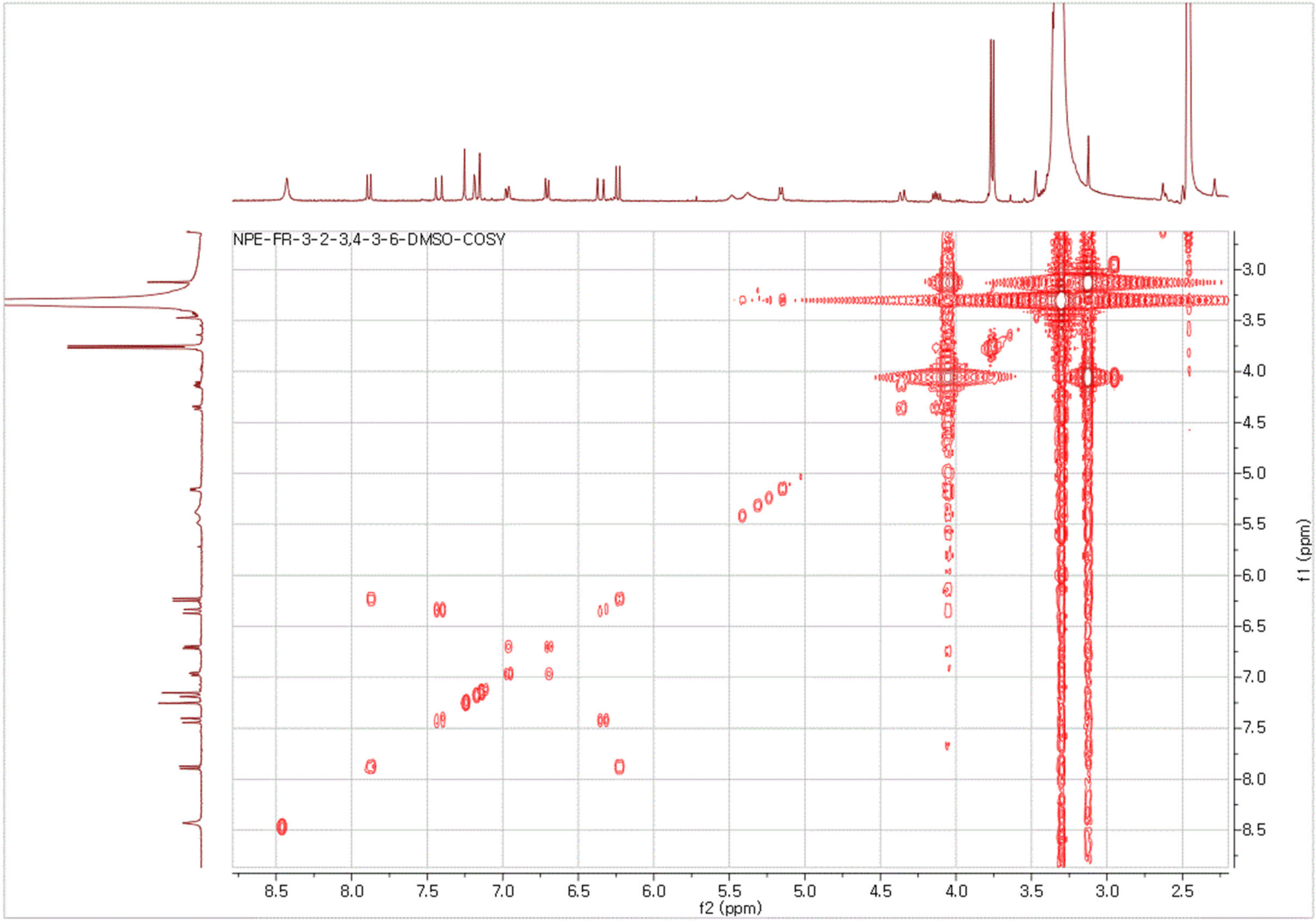

Figure S16. HSQC spectrum of **6** in DMSO-d_6

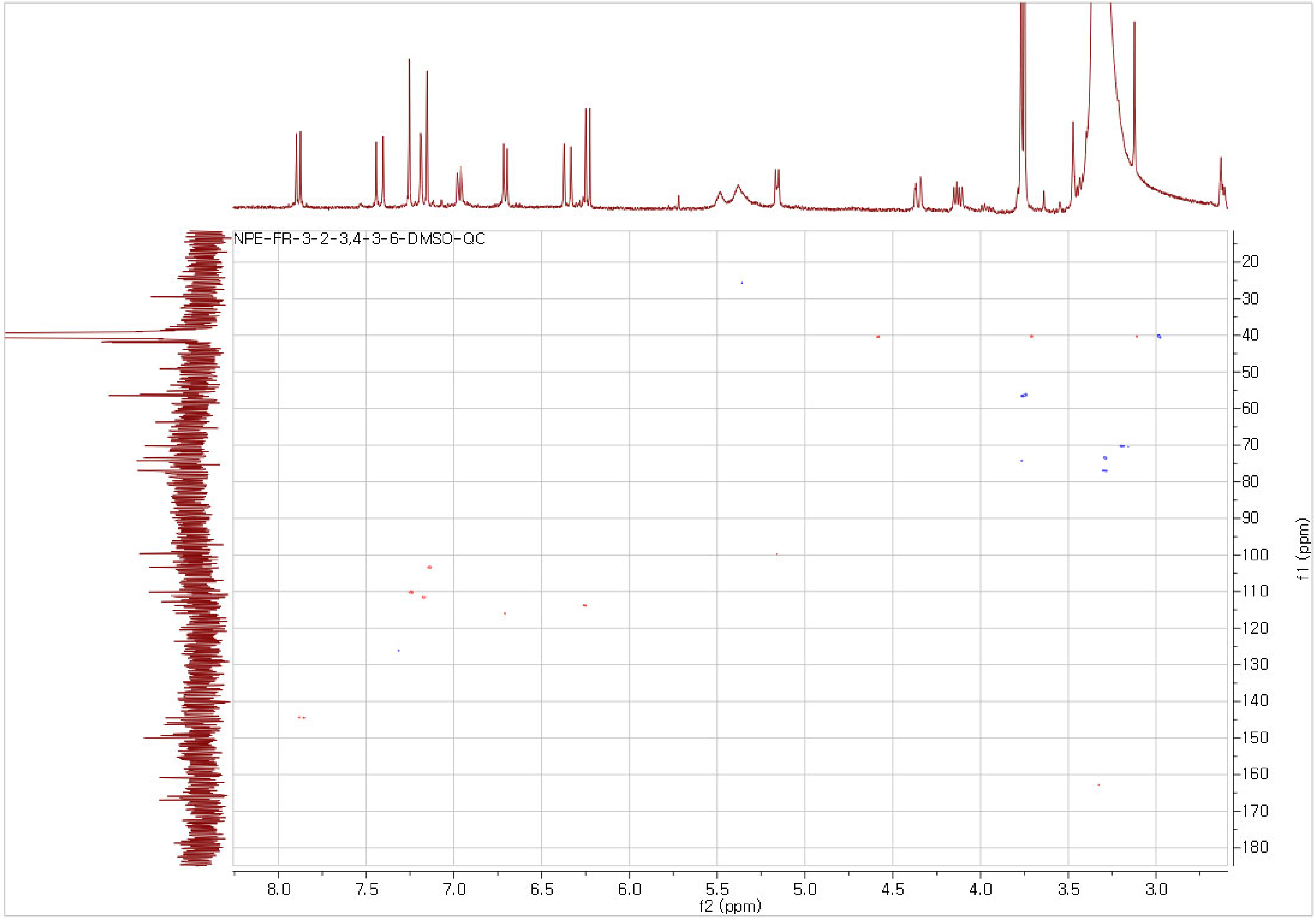

Figure S17. HMBC spectrum of **6** in DMSO-d_6

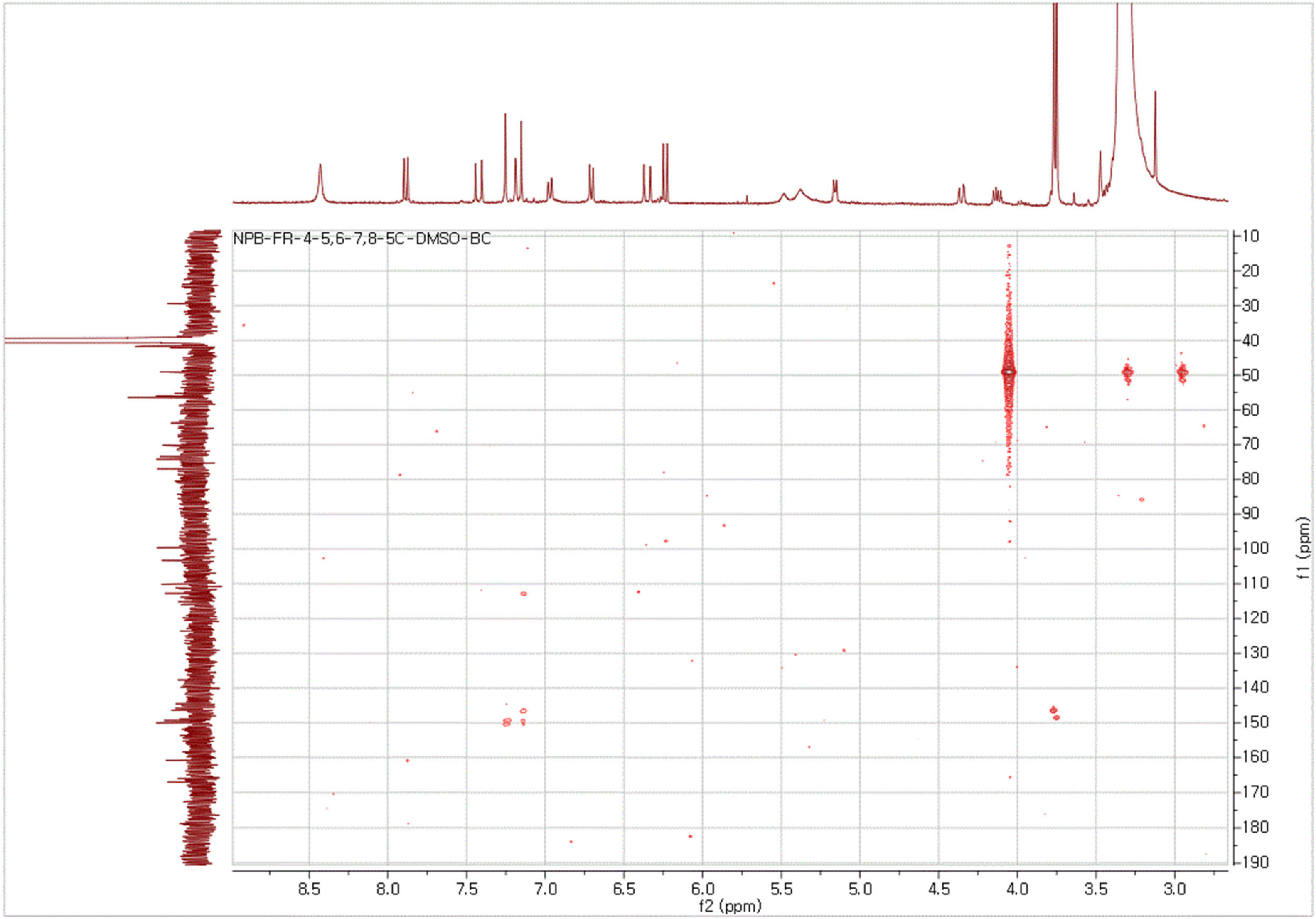

Figure S18. UV/PDA spectrum of **6**

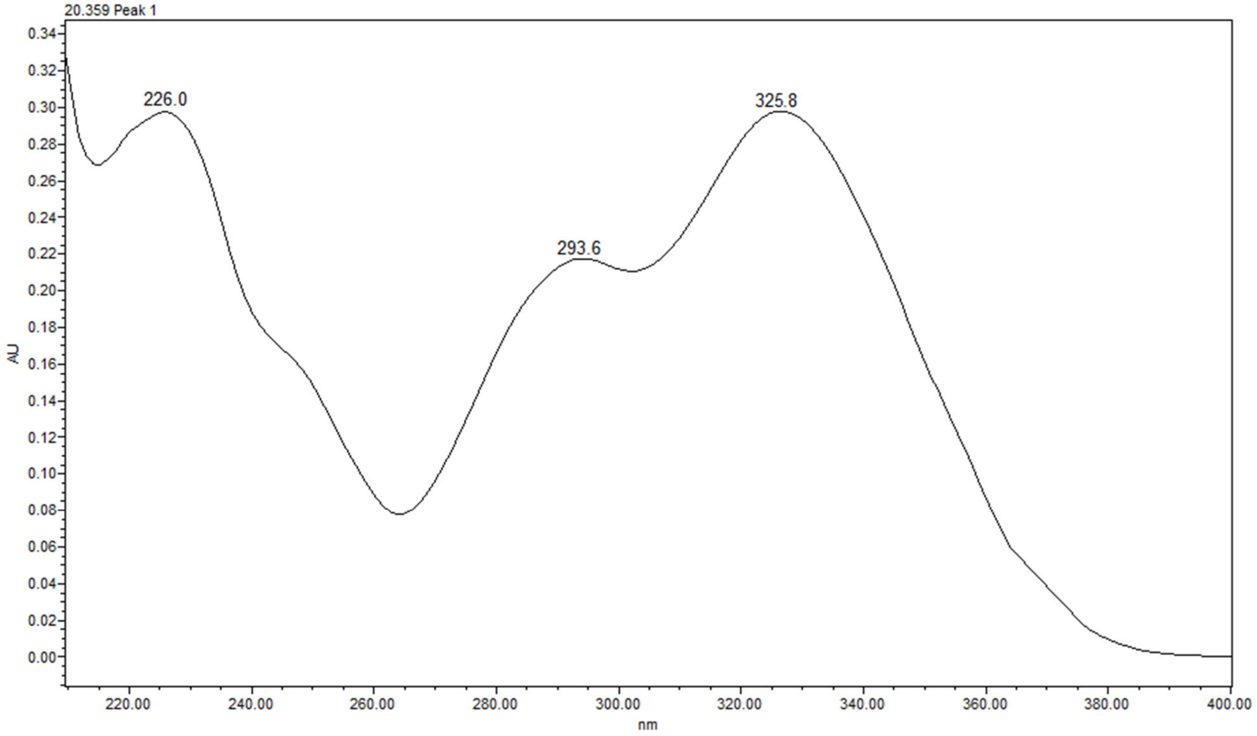

Figure S19. Wound healing area of compounds isolated from *N. peltata*

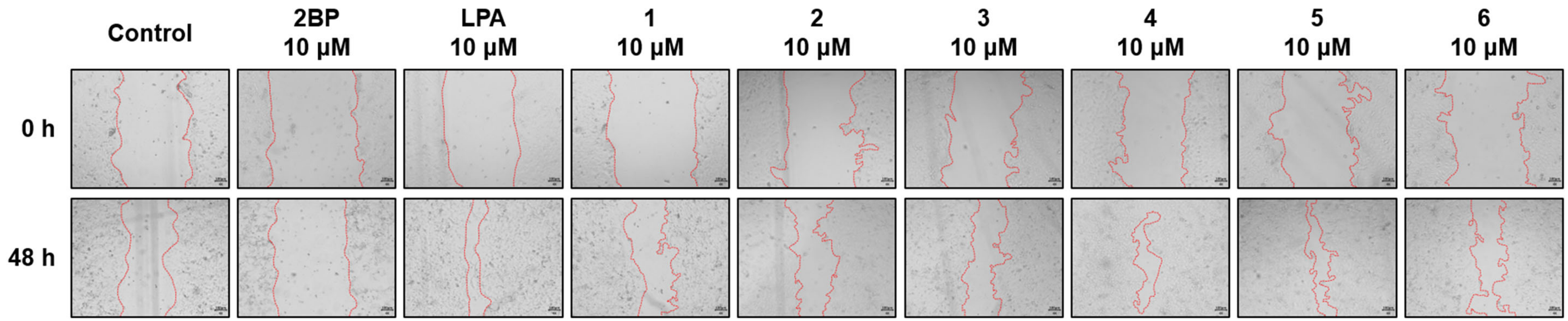

Supplement: Supplementary file 1 [file ijms-25-01268-s001.zip › ijms-2802781-supplementary.pdf]
